# Mitochondrial protein, TBRG4, modulates KSHV and EBV reactivation from latency

**DOI:** 10.1371/journal.ppat.1010990

**Published:** 2022-11-23

**Authors:** Huirong Zhang, Jason P. Wong, Guoxin Ni, Patricio Cano, Dirk P. Dittmer, Blossom Damania

**Affiliations:** Department of Microbiology and Immunology and Lineberger Comprehensive Cancer Center, and Center for AIDS Research, University of North Carolina at Chapel Hill, Chapel Hill, North Carolina, United States of America; Florida State University, UNITED STATES

## Abstract

Kaposi’s sarcoma-associated herpesvirus (KSHV) and Epstein-Barr (EBV) are gammaherpesviruses associated with multiple human malignancies. KSHV is the etiological agent of Kaposi’s Sarcoma, primary effusion lymphoma (PEL) and multicentric Castleman’s disease (MCD). EBV is associated with Burkitt’s lymphoma (BL), Hodgkin’s lymphoma (HL), nasopharyngeal carcinoma (NPC) and gastric carcinoma (GC). KSHV and EBV establish life-long latency in the human host with intermittent periods of lytic reactivation. Here, we identified a cellular factor named transforming growth factor-beta regulator 4 (TBRG4) that plays a role in the gammaherpesvirus lifecycle. We find that TBRG4, a protein that is localized to the mitochondria, can regulate lytic reactivation from latency of both KSHV and EBV. Knockdown of TBRG4 in cells latently infected with KSHV or EBV induced viral lytic gene transcription and replication. TBRG4 deficiency causes mitochondrial stress and increases reactive oxygen species (ROS) production. Treatment with a ROS scavenger decreased viral reactivation from latency in TBRG4-depleted cells. These data suggest that TBRG4 serves as a cellular repressor of KSHV and EBV reactivation through the regulation of ROS production.

## Author summary

Kaposi’s sarcoma-associated herpesvirus (KSHV) and Epstein-Barr (EBV) are double-stranded DNA tumor viruses that are associated with multiple cancers in the human population. These include both B and T cell lymphomas, as well as epithelial- and endothelial-derived cancers. Both EBV and KSHV exhibit two phases of their lifecycle, latency and lytic replication. During lytic reactivation, the virus switches from latency and replicates its viral genome to produce new infectious progeny. Here we report that the mitochondrial protein, TBRG4, serves as a cellular repressor of KSHV and EBV lytic reactivation through the regulation of ROS production.

## Introduction

Kaposi’s sarcoma-associated herpesvirus (KSHV, also known as HHV8) and Epstein-Barr virus (EBV) are members of the human gammaherpesvirus family and both are the etiological agents of several human malignancies [1–5]. Like other herpesviruses, KSHV and EBV exhibit a two-phase life cycle: latency and lytic replication. During latency, expression of viral genes is limited to only a subset of latency-associated transcripts, and no infectious virions are produced. The latent KSHV genome is replicated as circular episomes by the host cellular DNA polymerase. EBV is also maintained episomally in the nucleus of the host cell, and it establishes distinct types of latency based on specific latent gene expression patterns [6]. KSHV and EBV latency can be disrupted by physiological factors or exogenous stimuli. During viral reactivation, all viral genes are expressed, viral DNA is amplified, and progeny virions are produced [7,8]. Although lytic replication triggers host cellular antiviral responses, certain host cellular signaling pathways are co-opted by the virus to facilitate the assembly and release of infectious mature viral particles [9–11]. Thus, lytic reactivation of KSHV and EBV is a complex process involving a combination of both viral and cellular factors.

Several environmental factors have been reported to reactivate KSHV from latency, such as immunosuppression, viral co-infection, hypoxia, inflammatory cytokines, and oxidative stress [12]. Oxidative stress is a phenomenon caused by an accumulation of reactive oxygen species (ROS). AIDS-related KS tumors display high levels of oxidative stress and KSHV infection of endothelial cells induces oxidative stress [13,14]. Additionally, oxidative stress has been demonstrated to be involved in KSHV reactivation [15]. Hydrogen peroxide (H_2_O_2_), a reactive oxygen species, induces the expression of KSHV lytic genes in BCBL1 and BC-1 cells. Treatment with H_2_O_2_ induces the promoter activity of a key KSHV lytic switch protein, replication and transcription activator (RTA), whose expression is sufficient to initiate viral lytic reactivation. Inhibiting expression of ROS scavenging enzymes, such as catalase, also increases the expression of KSHV lytic transcripts [16]. In addition, hypoxia- and pro-inflammatory cytokine-induced KSHV reactivation is mediated by ROS [15]. The increased levels of ROS is also observed during EBV infection, and oxidative stress leads to EBV lytic reactivation and the increase of viral gene expression and/or induction of cellular signaling pathways [11,17,18].

TBRG4 (transforming growth factor beta-regulator 4, also known as FASTKD4) is a member of the Fas-activated serine/threonine kinase (FASTK) family which includes FASTK and its homologs FASTKD1-5. The structure of FASTKD proteins contains an N-terminal mitochondrial localization signal (MLS), two fast kinase-like domains (FAST_1 and FAST_2) of unknown function, and a C-terminal RNA-binding domain named RAP. FASTKD proteins are involved in the regulation of mitochondrial RNA biology, including RNA processing, maturation and protein translation, as well as modulation of mitochondrial respiration. TBRG4 maintains the mRNA level of mitochondrial proteins, ND3 and COX1, and modulates the processing of mitochondrial ND5-CYTB transcript [19,20]. Recently, it was reported that TBRG4 deficiency promoted apoptosis and increased ROS production [21,22].

Here, we report that the mitochondrial localized protein TBRG4 is important for inhibiting the switch from KSHV latency to lytic replication. Knockdown of TBRG4 in cells latently infected with KSHV initiated viral lytic gene transcription and induced viral replication. The induction of KSHV reactivation in TBRG4-silenced cells was further elevated after inducing expression of the lytic switch protein RTA or treatment with chemical inducers of lytic reactivation such as 12-O Tetradecanoyl-phorbol-13-acetate (TPA) and sodium butyrate (NaB). Depletion of TBRG4 increased ROS production, while treatment with a ROS scavenger significantly decreased the induction of KSHV lytic reactivation in TBRG4-depleted cells. Moreover, TBRG4 also regulated the EBV switch from latency to lytic reactivation. Knockdown of TBRG4 in latent EBV-infected cancer cell lines induced expression of EBV genes and increased viral genome copy number. These findings suggest that TBRG4 is a key regulator of KSHV and EBV reactivation, and that its expression is required for maintenance of viral latency and inhibition of viral lytic reactivation.

## Results

### Knockdown of TBRG4 expression increases KSHV lytic reactivation

The iSLK.219 cell line contains a recombinant KSHV that expresses a constitutive GFP marker and an RFP marker driven by a lytic cycle-specific promoter, which can be used as an indicator for lytically reactivated cells. The cells also have a doxycycline (Dox)-inducible RTA expression construct, allowing precise induction of KSHV lytic reactivation with doxycycline treatment. We found increased RFP signal from TBRG4 siRNA-treated cells compared to non-specific (NS) siRNA-treated cells at 36 and 48 h post-Dox induction (S1A and S1B Fig). This was the first indication that TBRG4 suppressed KSHV reactivation.

**Fig 1:**
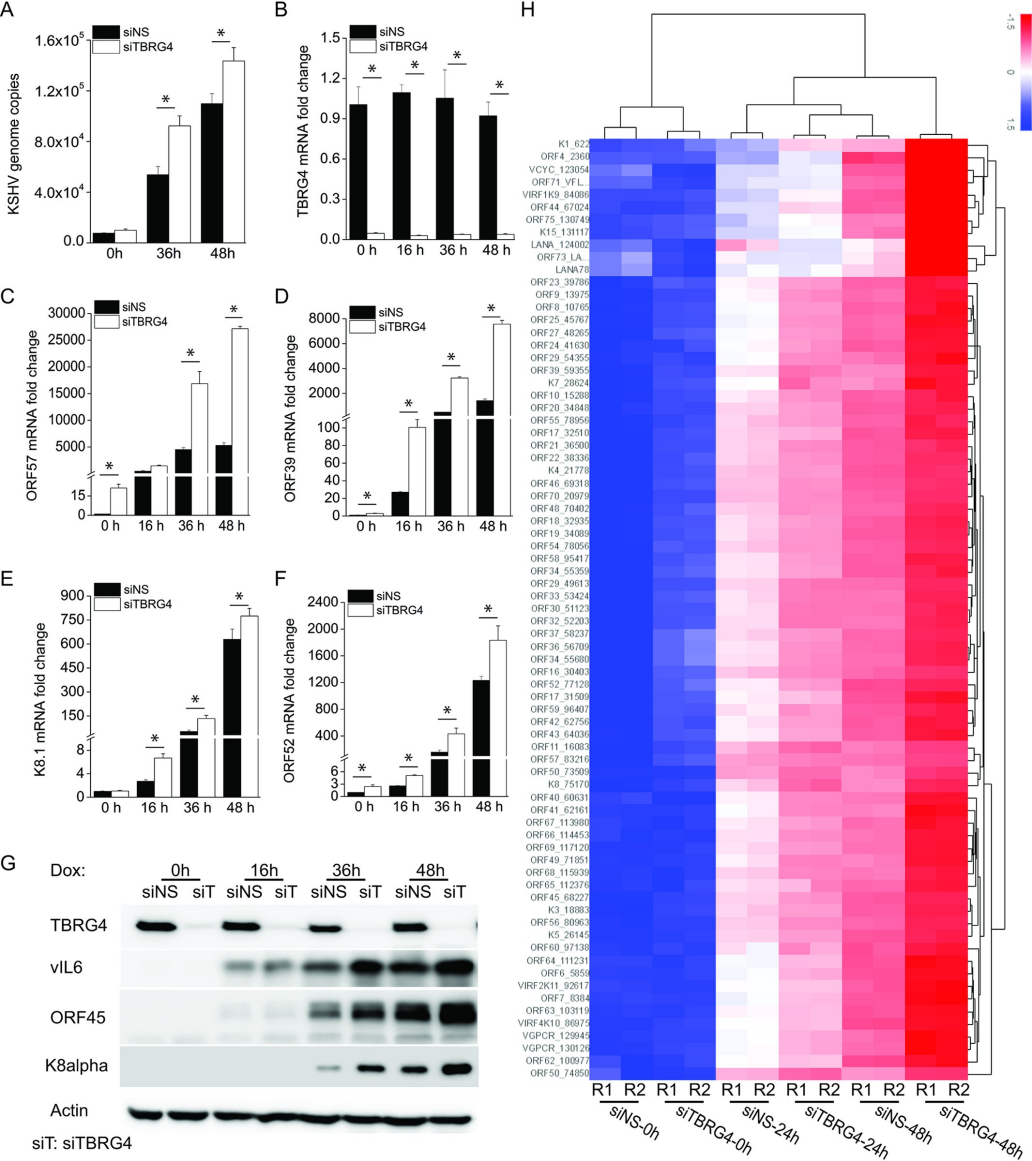
Knockdown of TBRG4 increases KSHV lytic reactivation. iSLK.219 cells were transfected with control non-specific (NS) siRNA or TBRG4 siRNA for 48 h and then treated with doxycycline (Dox; 0.2 μg/mL). **(A)** KSHV genome copy numbers in the supernatant were measured by real-time PCR at the indicated time. **(B-F)** RNA was extracted from the cells, and the knockdown efficiency of TBRG4 and the mRNA expression levels of the KSHV ORF57, ORF39, K8.1, and ORF52 genes were measured by real-time PCR. Target mRNA expression was normalized to β-actin mRNA and presented as fold induction. **(G)** Cell lysates were collected at the indicated time points, and western blots were performed with the indicated antibodies. **(H)** RNA was extracted from duplicate samples at the indicated time points, and KSHV viral transcript expression was measured by a KSHV real-time PCR-based array targeting 82 KSHV viral genes. Target viral mRNA expression levels were normalized to the mRNA level of cellular housekeeping genes to generate delta CT as a measure of relative expression. A heatmap for the KSHV viral array using the delta CT value was shown. Higher transcript levels are indicated by red, and lower levels by blue. Viral genes are listed on the vertical axis and samples on the horizontal. R1 and R2 indicate data from duplicate samples. The data shown are representative of three independent experiments. Data are presented as mean ± SD. *, P<0.05 by student’s t-test.

Consistent with these data, there were more KSHV genome copies in the supernatant of TBRG4 siRNA-treated cells than in control NS siRNA-treated cells after reactivation (Fig 1A). The knockdown efficiency of TBRG4 was confirmed by real-time PCR, and knockdown of TBRG4 did not affect the expression of other FASTK family genes (Figs 1B and S1C). We next measured the mRNA and protein levels of several viral genes to determine the impact of TBRG4 on KSHV gene expression. We found that TBRG4-silenced cells displayed an increase in the mRNA expression of KSHV genes ORF57 (an immediate-early gene), ORF39 (an early gene), as well as K8.1 and ORF52 (late genes) (Fig 1C–1F). The expression level of viral proteins, viral interleukin-6 (vIL-6) (a latent and lytic gene), ORF45 (an immediate-early gene), and K8α (an early gene) was also higher in TBRG4-deficient cells compared with control cells (Fig 1G). In sum, by three orthogonal measures, genome copy number, viral mRNA transcription, and viral protein expression, TBRG4 inhibited viral reactivation.

To broadly profile KSHV viral gene expression in TBRG4-silenced cells compared to control cells, we performed real-time PCR-based transcriptional profiling of the KSHV viral genome. As seen in Fig 1H and S1 Table, Dox treatment successfully induced KSHV gene expression, and depletion of TBRG4 increased the transcriptional level of viral genes at 24 and 48 h post-Dox reactivation, which correlates with our qRT-PCR data (Fig 1C–1F). These data indicate that TBRG4 plays a negative regulatory role during KSHV lytic reactivation rather than having a more limited effect on selected lytic viral genes.

### Depletion of TBRG4 switches KSHV from latency into lytic replication

**Fig 2:**
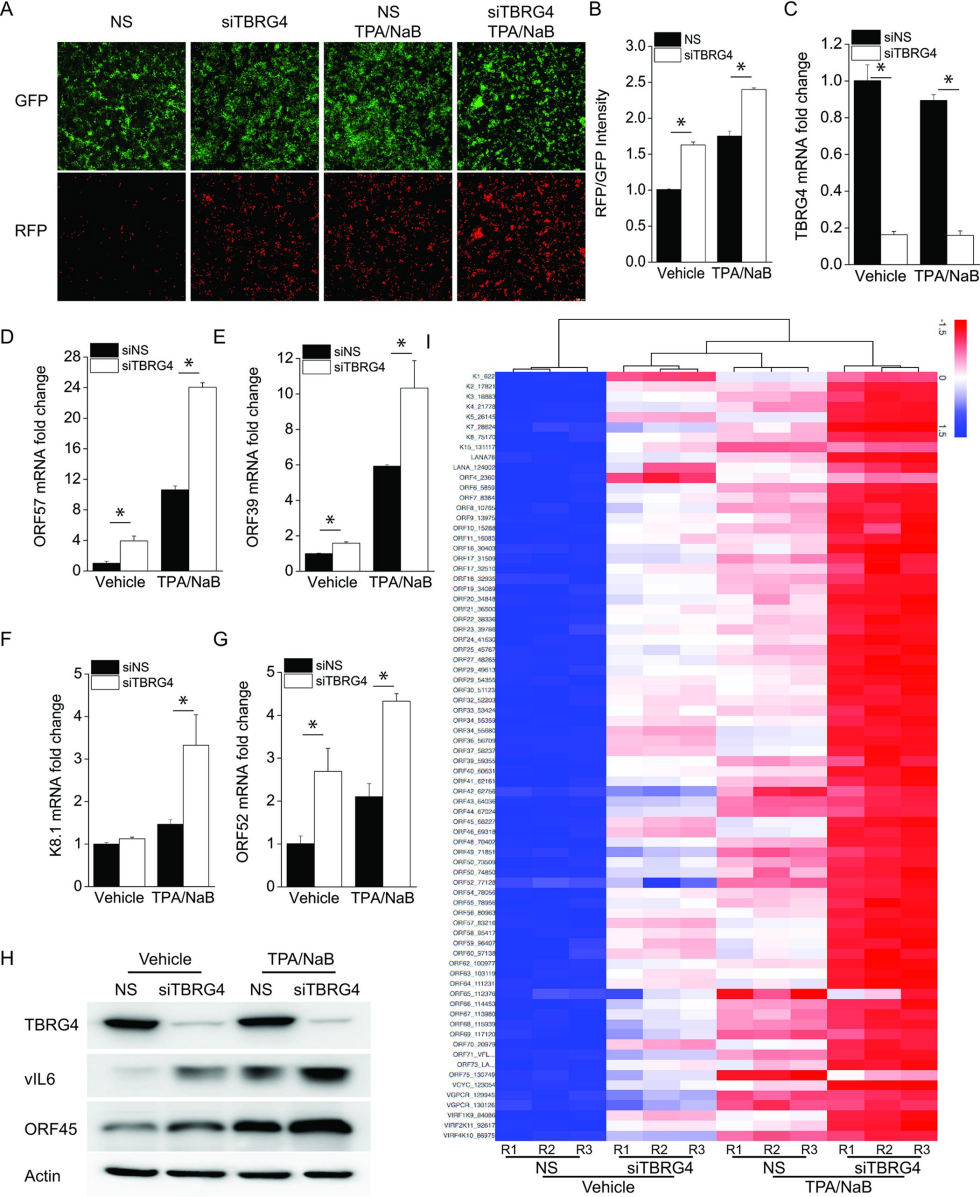
Depletion of TBRG4 switches KSHV from latency into lytic reactivation. KSHV-293 cells were transfected with NS or TBRG4 siRNA for 72 h. KSHV-293 cells were then reactivated with TPA (25 ng/ml) and NaB (1 mM) for 36 h. **(A)** RFP- and GFP-positive cells were imaged at 0 and 36 h post reactivation. **(B)** RFP and GFP intensities were monitored and quantitated by Leica DMi8. **(C-G)** RNA was extracted from the cells, and the knockdown efficiency of TBRG4 and mRNA expression level of KSHV ORF57, ORF39, K8.1 and ORF52 genes were measured by real-time PCR, and the fold change was normalized to β-actin mRNA. **(H)** Cells were harvested and western blots were performed with the indicated antibodies. **(I)** KSHV-293 cells were treated as described in the text. RNA was extracted from triplicate samples and KSHV viral transcript expression was measured by a KSHV real-time PCR-based array. Target viral mRNA expression levels were normalized to the mRNA level of cellular housekeeping genes to generate delta CT and are shown in a heatmap. R1, R2, and R3 indicate data from triplicate samples.

We next used KSHV-293 cells to confirm the effect of TBRG4 on regulating the KSHV latent-lytic switch. Similar to iSLK.219 cells, the KSHV-293 cell line contains a recombinant KSHV that constitutively expresses a GFP marker and expresses an RFP marker from a KSHV lytic promoter, but lacks Dox-inducible RTA. Chemical inducers, such as TPA and NaB (TPA/NaB) are used to induce RTA from its endogenous promoter, which then triggers complete KSHV viral lytic reactivation. We used TPA/NaB to induce KSHV lytic reactivation in KSHV-293 cells transfected with control or TBRG4 siRNAs. The TBRG4-depleted cells exhibited a much higher RFP signal than NS control cells, similar in magnitude to the signal observed in TPA/NaB-treated NS cells (Fig 2A). Furthermore, the RFP signal was enhanced in TPA/NaB-treated TBRG4-depleted cells compared to TPA/NaB-treated NS control cells (Fig 2A). The RFP and GFP intensities in the cells were further quantified as fold induction of the RFP/GFP ratio (Fig 2B). The knockdown efficiency of TBRG4 was confirmed by real-time PCR (Fig 2C). We observed that the mRNA expression of KSHV genes ORF57, ORF39, K8.1, and ORF52, was increased in comparison to NS control cells upon TPA/NaB treatment (Fig 2D–2G). Consistent with these data, the viral gene profile demonstrated that the majority of viral genes were upregulated in TBRG4-depleted cells compared to NS control cells in the vehicle control samples (Fig 2I). Furthermore, when the cells were induced with TPA/NaB, the depletion of TBRG4 induced viral gene expression even further compared to the NS control cells (Fig 2I and S2 Table). These data indicate that depletion of TBRG4 broadly activates the transcription of viral genes as the TBRG4-depleted cells exhibited a significantly higher RFP signal than NS control cells in the absence of chemical inducers, which was similar in magnitude to the signal observed in TPA/NaB-treated NS cells (Fig 2A). In addition, the expression of KSHV lytic proteins, vIL-6 and ORF45, was also induced in TBRG4-depleted cells (Fig 2H). This suggests that TBRG4 dampens sporadic KSHV reactivation in these cells. Taken together, these findings indicate that TBRG4 indeed contributes to maintaining KSHV latency and inhibiting TPA/NaB-induced viral lytic replication.

### Depletion of TBRG4 induces KSHV lytic reactivation in PEL cells

**Fig 3:**
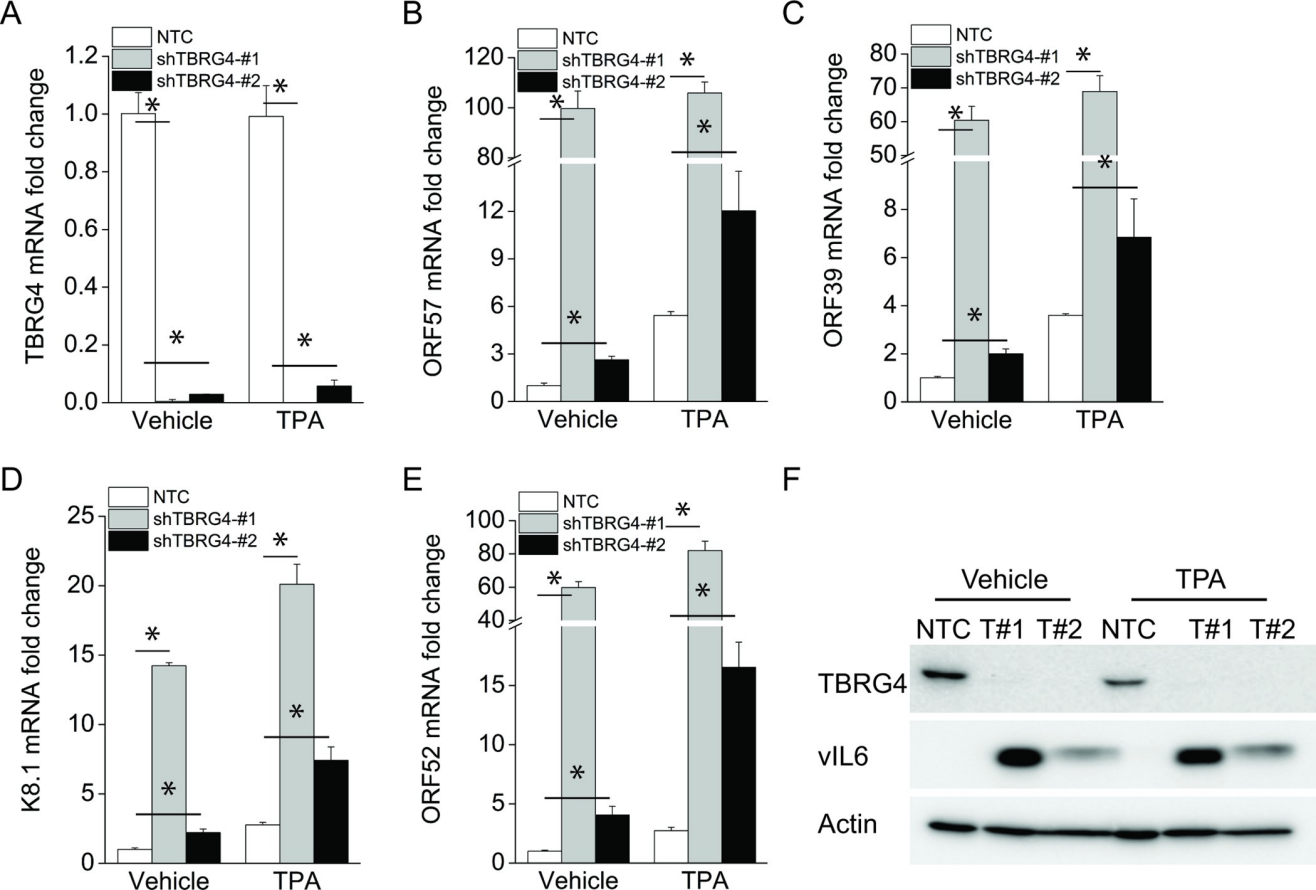
Suppression of TBRG4 induces KSHV lytic reactivation in PEL cells. BCBL1 cells were infected with lentivirus expressing a non-targeting shRNA, or two different shRNAs targeting TBRG4 for 5 d and then treated with 25 ng/ml TPA for 16 h. **(A-E)** RNA was extracted from the cells, and knockdown efficiency of TBRG4 and mRNA expression levels of the viral ORF57, ORF39, K8.1, and ORF52 was measured with real-time PCR and normalized to β-actin mRNA. **(F)** Cell lysates were collected and western blots were performed with indicated antibodies.

To further validate these observations, we explored the role of TBRG4 in PEL using BCBL1 cells, which were derived from a KSHV-infected patient. BCBL1 cells were infected with a control lentiviral shRNA or shRNAs targeting TBRG4 transcripts, and then KSHV lytic reactivation was induced with TPA for 16 hours to promote KSHV lytic reactivation. The knockdown efficiencies of both shTBRG4 #1 and shTBRG4 #2 were measured by real-time PCR (Fig 3A), with shTBRG4 #1 showing better efficiency than shTBRG4 #2. Similar to what we observed in KSHV-293 cells, the transcript levels of several viral genes ORF57, ORF39, K8.1, and ORF52 were significantly induced in TBRG4-depleted KSHV latently infected BCBL1 cells compared to non-targeting control (NTC)-treated cells. These data indicate that TBRG4 controls spontaneous KSHV reactivation in PEL. The expression of viral genes was also elevated in TBRG4-depleted cells compared to control cells after TPA-induced lytic reactivation (Fig 3B–3E). Consistent with these data, the expression level of viral vIL-6 protein was also increased in TBRG4-depleted cells compared to NTC-treated cells (Fig 3F). These data demonstrate that TBRG4 contributes to the KSHV latent-lytic switch in PEL.

### Increased editing of the KSHV Kaposin transcript in TBRG4-depleted cells

We previously reported that KSHV lytic reactivation was mediated by ADAR1, which is the enzyme involved in adenosine-to-inosine (A-to-I) RNA editing in mammalian cells and that the A-to-I editing of KSHV Kaposin transcript is also affected by ADAR1. Furthermore, the level of A-to-I editing in Kaposin RNA is increased during KSHV lytic reactivation [23]. Since inosine (I) is recognized as guanosine (G) during protein translation, A-to-I editing is also known as A-to-G editing. We found that there was a higher proportion of edited G in the edited site of the Kaposin RNA in TBRG4-depleted iSLK.219 cells compared to control cells (S2A Fig). To determine whether TBRG4 contributes to A-to-G editing activity, we co-transfected A-to-G editing luciferase reporter plasmid with an expression plasmid of TBRG4 or ADAR1 (as a positive control). In the A-to-G editing reporter plasmid, the stop codon (UAG) prevents Renilla luciferase expression, whereas an A-to-G editing event within the stop codon leads to Renilla luciferase expression (S2B Fig). Renilla luciferase activity was increased by expression of ADAR1, the primary A-to-G editing enzyme in mammalian cells. However, the level of luciferase activity was not changed in Flag-tagged TBRG4-overexpressing cells, indicating that TBRG4 had no effect on the activity of A-to-G editing (S2C Fig). We previously reported that the level of A-to-G editing in Kaposin RNA is increased during KSHV lytic reactivation [23]. As shown in S2A Fig, the unedited nucleotide (A) of Kaposin transcripts was the predominant nucleotide in latently infected iSLK.219 cells, while a large proportion of edited G was observed in the lytically reactivated cells. Kaposin transcript is expressed in KSHV latency and is induced during lytic reactivation, the expression of Kaposin transcript was also increased in TBRG4-depleted cells compared to control cells (S2D Fig). Taken altogether, these data imply that the increased G editing in TBRG4-depleted cells was caused by the induction of KSHV lytic reactivation due to TBRG4 knockdown and that TBRG4 does not directly modulate the RNA editing of the Kaposin transcript.

### Mitochondrial localization signal (MLS) domain and the RNA binding RAP domain of TBRG4 contribute to the inhibition of KSHV lytic reactivation

TBRG4 contains an MLS domain in the N-terminal and a RAP domain in the C-terminal (S3A Fig). To identify whether these TBRG4 domains are associated with the inhibition of KSHV lytic reactivation, we transfected iSLK.219 cells with plasmids expressing full-length TBRG4, TBRG4-ΔMLS, TBRG4-ΔRAP or empty vector control (S3B Fig). Consistent with the knockdown experiments, the expression of viral mRNA was most significantly inhibited in cells overexpressing full-length TBRG4 compared to cells transfected with the control vector, and to a lesser extent by the TBRG4-ΔMLS and TBRG4-ΔRAP proteins (S3C–S3F Fig). These data suggest that both MLS and RAP domains are required for TBRG4 to inhibit KSHV lytic reactivation.

### Depletion of TBRG4 causes mitochondrial stress and increases ROS production

**Fig 4:**
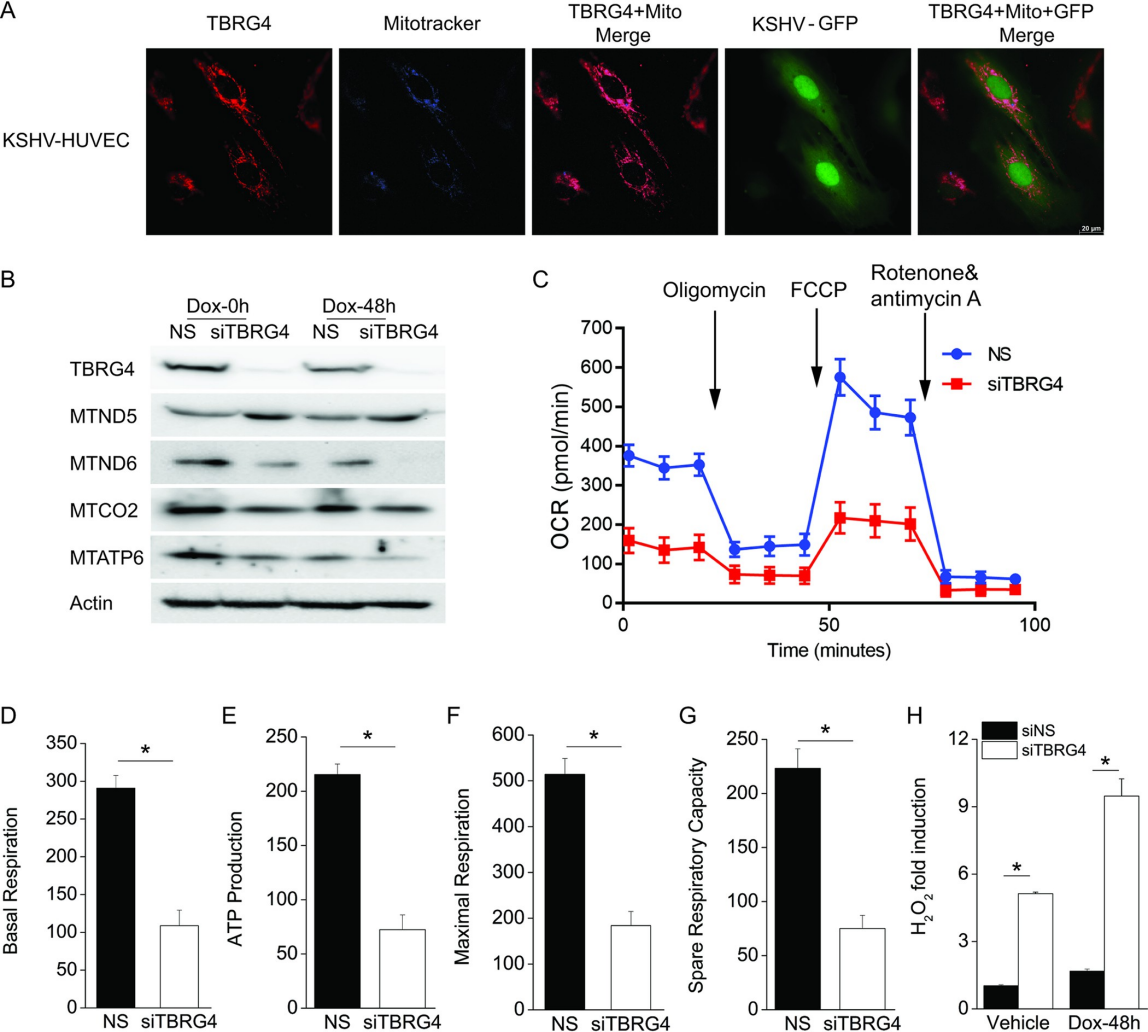
Depletion of TBRG4 causes mitochondrial stress and increases ROS. **(A)** KSHV-HUVEC were treated with MitoTracker to visualize the mitochondria (blue), then fixed and stained with TBRG4 antibody (red). KSHV-HUVEC cells contain a recombinant KSHV constitutively expressing GFP (green). Images were collected using a Leica DMi8. **(B)** iSLK.219 cells were transfected with NS and TBRG4 siRNA for 48 hours and then treated with 0.2 μg/ml Dox for 0 and 48 h. Cells lysates were collected and western blots were performed with the indicated antibodies. **(C-G)** iSLK.219 cells were transfected with NS and TBRG4 siRNA for 96 h, then mitochondrial respiration was evaluated in living cells using a Seahorse XF analyzer. Oxygen consumption ratio (OCR) was measured during sequential treatment with oligomycin (1.5 μM), FCCP (0.5 μM) and rotenone/antimycin A (1 μM). Quantification of the basal oxygen consumption, ATP production, maximal respiration, and spare respiratory capacity are shown in the indicated panels. **(H)** iSLK.219 cells were transfected with NS and TBRG4 siRNA for 48 h, then treated with 0.2 μg/ml Dox for 0 and 48 h. The level of H_2_O_2_ was measure with ROS-Glo H_2_O_2_ assay. H_2_O_2_ substrate was added for 5 h and luminescence signal was measured and is presented as fold induction.

To gain insight into potential mechanisms by which TBRG4 might suppress the switch from KSHV latency to lytic reactivation, we investigated the subcellular localization of TBRG4 in KSHV-infected HUVEC cells, which are flat and have a large cytoplasm. We also checked TBRG4 localization in uninfected iSLK cells compared to iSLK.219. As shown in Figs 4A and S4A, TBRG4 was localized to the mitochondria in both KSHV-HUVEC cells and uninfected iSLK cells. Since the RNA binding domain named RAP is required for TBRG4 to regulate KSHV lytic reactivation, we performed a mitochondrial RNA (mtRNA) array to identify whether TBRG4 affects the expression of these transcripts. We found that TBRG4 depletion did not significantly affect the expression of mtRNA (S4B Fig), but its depletion did change the expression of mtRNA translated proteins MTND5, MTND6, MTCO2, and MTATP6 relative to control cells (Fig 4B). Since all mtDNA-encoded proteins are components of the oxidative phosphorylation (OXPHOS) system/respiratory chain, we investigated the efficiency of OXPHOS by measuring oxygen consumption rates (OCR) in TBRG4-depleted cells and control cells. We found that TBRG4-depleted cells displayed significantly decreased key parameters of mitochondrial function (such as basal respiration, ATP production, maximal respiration, and spare respiratory capacity) compared to control cells (Fig 4C–4G). We also found that levels of cellular H_2_O_2_, a ROS, was significantly increased in TBRG4-silenced cells, which can be caused by mitochondrial dysfunction (Fig 4H). It has been reported that H_2_O_2_ induces promoter activity of ORF50/RTA which is a key regulator of the KSHV lytic cycle [16]. Hence, we next tested the impact of TBRG4 depletion on KSHV ORF50 promoter activity. We found that KSHV ORF50 promoter activity was increased in TBRG4-depleted cells compared to control cells (S4C Fig). Taken together, these data suggest that the mitochondrial protein, TBRG4, affects the function of oxidative phosphorylation and subsequent ROS generation and this results in activation of the KSHV ORF50 promoter.

### ROS scavenger treatment affects KSHV lytic reactivation from latency in TBRG4-depleted cells

**Fig 5:**
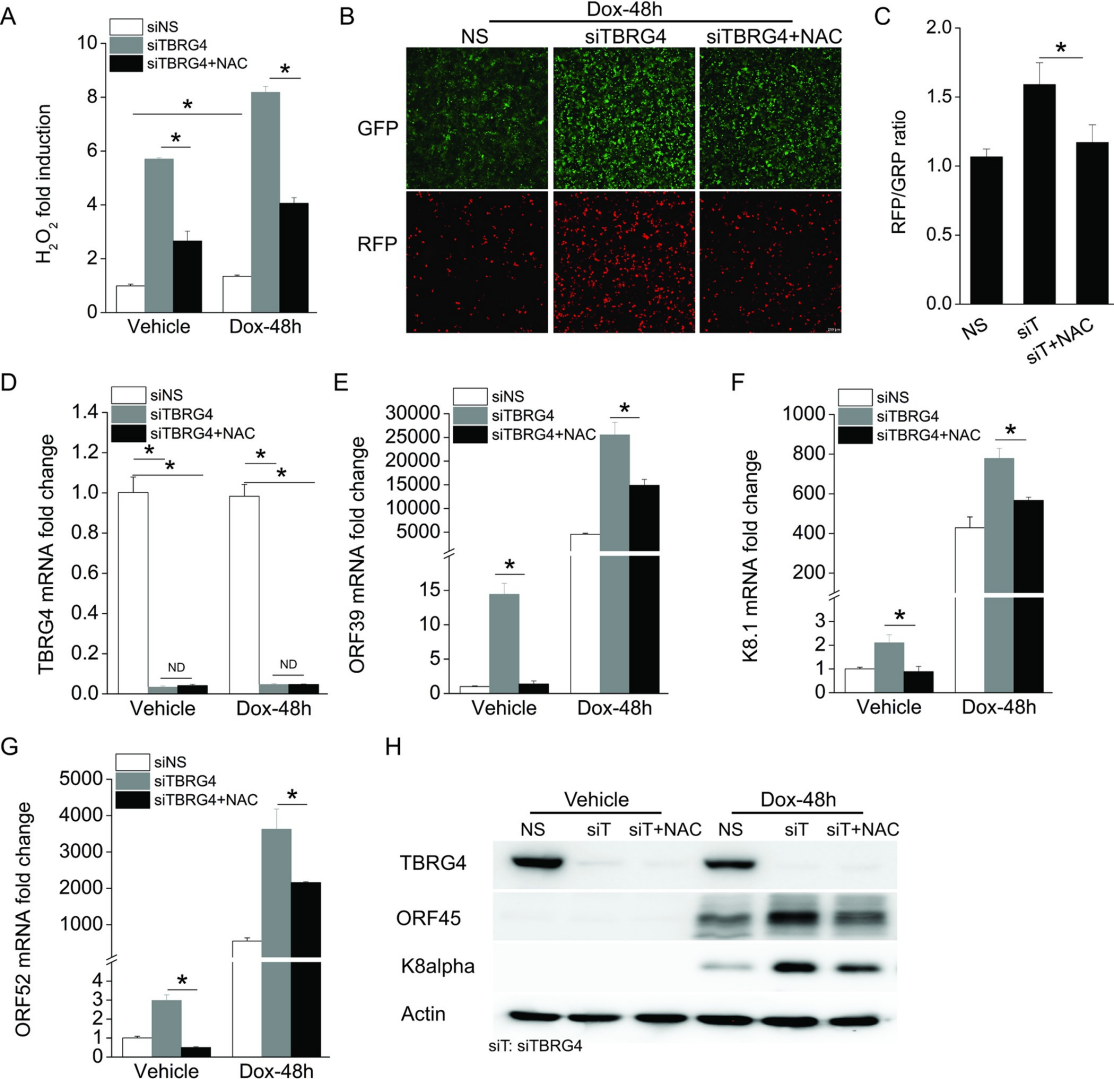
A ROS scavenger affects KSHV lytic reactivation from latency in TBRG4-depleted cells. iSLK.219 cells were transfected with NS, siTBRG4 or siTBRG4 along with 2 mM NAC for 48 h and then treated with Dox for 48 h. **(A)** The level of H_2_O_2_ was measured with ROS-Glo H_2_O_2_ assay. H_2_O_2_ substrate was added for 5 h and luminescence signal was measured and presented as fold induction. **(B)** GFP- and RFP- positive cells were imaged at 48 h post-Dox treatment. **(C)** GFP and RFP intensities were measured using a Clariostar plate reader, and the RFP/GFP ratio was calculated at 48 hours post-Dox treatment. **(D-G)** The mRNA expression of TBRG4, ORF39, K8.1 and ORF52 was measured by real-time PCR. **(H)** Western blots were performed with the indicated antibodies.

Since TBRG4 depletion resulted in upregulation of ROS and KSHV ORF50 promoter activation, we next examined its impact on lytic reactivation. To identify whether the increase of ROS in TBRG4-depleted cells leads to KSHV lytic reactivation, we used a ROS scavenger N-acetyl-L-cysteine (NAC) to inhibit ROS production in TBRG4-silenced iSLK.219 cells. Intracellular H_2_O_2_ was significantly increased in TBRG4-depleted cells compared to control cells, and treatment with NAC significantly decreased the level of H_2_O_2_ induction in TBRG4-depleted cells (Fig 5A). As described above and shown in S1 Fig, depletion of TBRG4 significantly increased RFP signal (an indicator of KSHV lytic reactivation) compared to control cells. However, treatment of TBRG4-depleted cells with NAC significantly decreased the level of RFP signal to a level similar to that in control cells (Fig 5B and 5C). The knockdown efficiency of TBRG4 was not affected by NAC treatment (Fig 5D and 5H).

A similar effect was observed using KSHV transcription as a readout. The transcription of KSHV genes was induced in TBRG4-depleted cells at 0 and 48 hours post reactivation, while treatment with NAC significantly decreased the induction of these viral lytic genes compared to untreated TBRG4-depleted cells (Fig 5E–5G). Consistent with this data, the expression level of KSHV proteins, K8α and ORF45, also decreased with NAC treatment in TBRG4-silenced cells at 48 hours post reactivation (Fig 5H). It has been reported that ROS induction during KSHV lytic reactivation is mediated through the activation of mitogen-activated protein kinase ERK1/2, JNK, and p38 pathways, and that the phosphorylation of ERK1/2 is required for KSHV lytic reactivation from latency [16,24]. To identify whether TBRG4-deficiency can further induce phosphorylation of ERK1/2 after ROS production, we measured the phosphorylation level of ERK1/2 in TBRG4-depleted and control cells. We found that the phosphorylation of ERK1/2 was increased in TBRG4-depleted cells compared to control cells (S4I Fig). Next, we treated TBRG4-depleted cells with an ERK inhibitor (U0126) and measured KSHV lytic reactivation. We found that treatment of TBRG4-depleted cells with U0126 significantly decreased the expression of KSHV genes compared to untreated TBRG4-depleted cells (S4D–S4I Fig). These data suggest that TBRG4-mediated suppression of lytic reactivation is associated with the ERK1/2 signaling pathway. Taken together, these data place ROS downstream of TBRG4 and suggests that TBRG4 suppresses KSHV reactivation through limiting ROS in latently infected cells.

### Knockdown of TBRG4 induces EBV lytic reactivation

**Fig 6:**
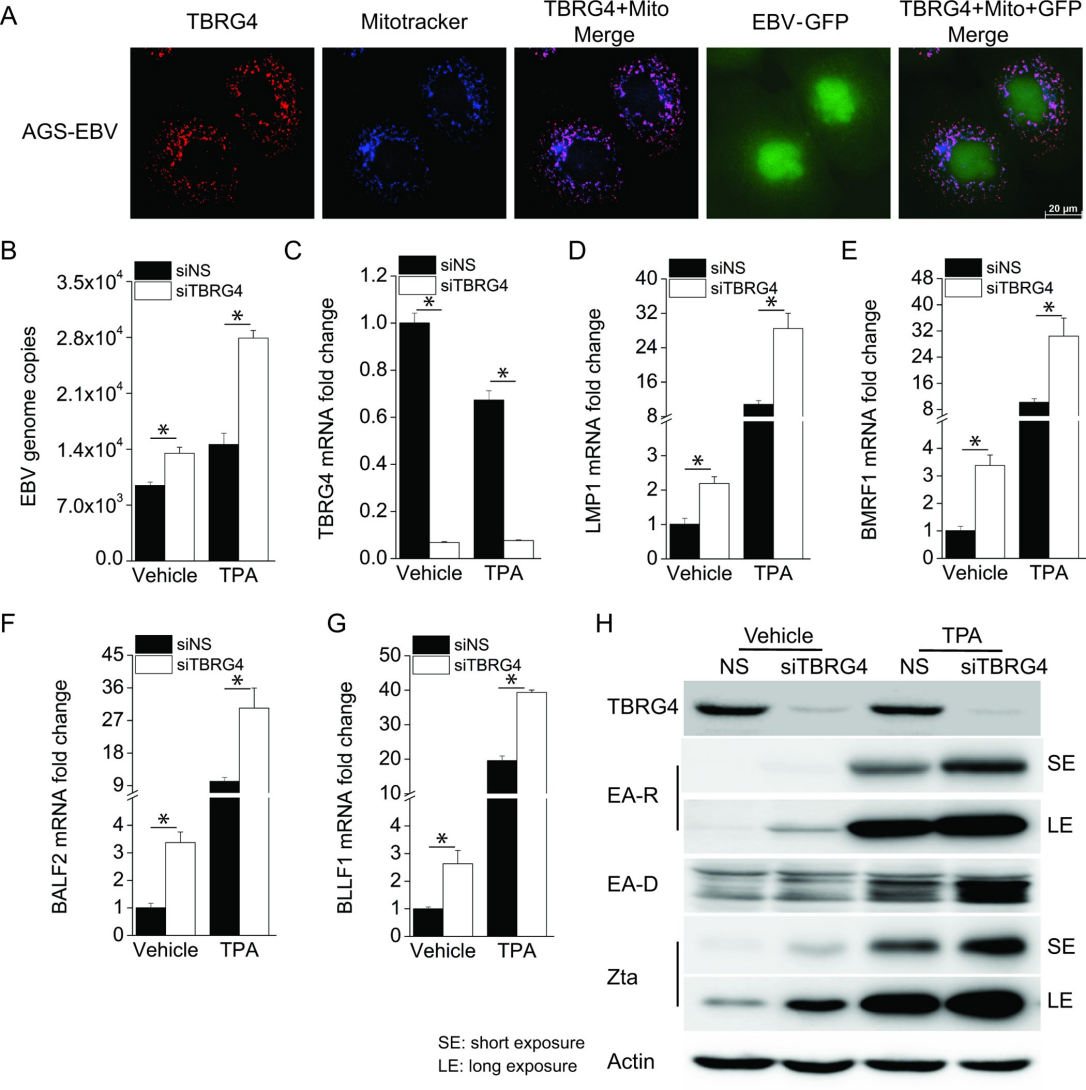
Knockdown of TBRG4 also induces EBV lytic reactivation. **(A)** AGS-EBV cells were treated with MitoTracker to visualize the mitochondria (blue), then fixed and stained with TBRG4 antibody (red). AGS-EBV cells contain a recombinant EBV constitutively expressing GFP (green). Images were taken using Leica DMi8. **(B)** AGS-EBV cells were transfected with control NS or TBRG4 siRNA for 48 h and then treated with TPA for 24 h. EBV genome copies were measured by real-time PCR in the supernatant from vehicle- and TPA-treated cells. **(C-G)** RNA was extracted from the AGS-EBV cells treated as in (B), and the knockdown efficiency of TBRG4 and the mRNA expression of EBV genes LMP1, BMRF1, BALF2, BLLF1 were measured by real-time PCR. **(H)** Cell lysates were harvested from the AGS-EBV cells treated as in (B), and western blots were performed with the indicated antibodies. The data shown are representative of three independent experiments. Data are presented as mean ± SD. *, P<0.05 by student’s t-test.

Since oxidative stress also plays a role in the regulation of EBV lytic reactivation [25,26], we next evaluated the function of TBRG4 in modulating EBV lytic reactivation. We performed similar experiments in an EBV latently infected gastric cancer cell line (AGS-EBV). The AGS-EBV cell line contains a recombinant EBV genome that constitutively expresses a GFP marker. We found that TBRG4 protein colocalized with the mitochondria in AGS-EBV cells (Fig 6A). EBV genome copies in the supernatant were significantly increased in TBRG4-depleted cells, to a level similar to that observed in the cells with TPA-induced lytic reactivation (Fig 6B). The knockdown efficiency of TBRG4 in AGS-EBV cells was measured with real-time PCR (Fig 6C). The mRNA levels of several EBV genes, such as LMP1 (latent gene), BMRF1 (immediate early gene), BALF2 (early gene), and BLLF1 (late gene), were significantly increased in TBRG4-depleted cells compared to control cells with and without TPA treatment (Fig 6D–6G). As seen with KSHV, TBRG4 depletion induced the spontaneous rates of viral reactivation (without TPA) and also increased the magnitude of TPA-induced reactivation (Fig 6B–6G). TBRG4-depleted cells showed induction and elevated expression of the EBV lytic proteins EA-D, EA-R, and Zta compared to control cells with or without TPA treatment (Fig 6H). A similar phenotype was observed in Akata-BX1 Burkitt lymphoma cells, where the expression of EBV genes was also increased in TBRG4-depleted cells compared to NTC-treated cells (S5 Fig). Taken together, these data (genome copy number, mRNA levels and protein levels) indicate that TBRG4 suppresses EBV lytic reactivation.

To identify whether TBRG4-mediated regulation of EBV lytic reactivation is also associated with ROS production, we did similar experiments in AGS-EBV cells. We found that knockdown of TBRG4 increased the level of ROS production in AGS-EBV cells (S6A and S6B Fig). Next, we used NAC to inhibit ROS production in TBRG4-depleted cells. As expected, treatment with NAC reduced ROS levels in TBRG4-depleted cells (S6A Fig). Moreover, NAC treatment of TBRG4-depleted cells also significantly decreased the expression of EBV genes compared to untreated TBRG4-depleted cells (S6C–S6G Fig). Therefore, TBRG4-depletion results in increased ROS production in AGS-EBV cells and also contributes to the induction of EBV lytic reactivation.

## Discussion

It has been shown that several cellular proteins can regulate the life cycle of KSHV, either directly, by affecting the functions of viral proteins, or indirectly, by altering the cellular environment. Ste20-like kinase (hKFC) and cellular poly (ADP-ribose) polymerase 1 (PARP-1) modify the viral activator RTA and repress RTA-mediated lytic gene transcriptional activation to maintain KSHV latency [27,28]. NLRX1 (NLR family member X1) and MAVS are localized in the mitochondria and facilitate KSHV replication through repression of the innate immune response during viral lytic reactivation [29,30]. Here, we identified another mitochondrial-localized protein, TBRG4, whose depletion induced the expression of KSHV lytic genes and increased the production of viral genomes. However, the mechanism of action for TBRG4 is new. Although depletion of TBRG4 did not decrease cell viability at earlier time points and only slightly decreased the cell viability in uninfected iSLK cells at later timepoints (S1D Fig), we found that suppression of TBRG4 did not modulate type I interferon (IFN) production in KSHV uninfected iSLK cells (S1E Fig). However, in KSHV-infected iSLK cells, when we knockdown TBRG4, there is an induction of interferon (S1F Fig) because the virus is reactivating and replicating in these cells to a greater extent due to the fact TBRG4 is depleted and hence the innate immune response to the virus kicks in and interferon is produced.

Increased amounts of ROS have been detected in both EBV and KSHV-infected cells compared to uninfected cells, and ROS have been implicated in playing a role in regulating lytic reactivation in both viruses [31,32]. EBV induces the production of ROS in Burkitt’s lymphoma cells through the actions of its proteins EBNA2 and LMP1, while targeting LMP1-mediated oxidative stress suppresses EBV lytic reactivation [33]. Both exogenous H_2_O_2_ and catalase inhibitor-induced endogenous H_2_O_2_ have been shown to promote the switch from KSHV latency to lytic reactivation in PEL and endothelial cells [16]. This study is consistent with prior work because a reduction of ROS in TBRG4-depleted cells by NAC treatment abolished the TBRG4 phenotype. Therefore, we suggest that TBRG4 promotes maintenance of KSHV and EBV latency and inhibits viral lytic reactivation through limiting ROS production.

Why would mitochondria play such an important role in controlling viral gene regulation? It is currently estimated that mitochondria contain at least 1500 different proteins. Approximately 99% of mitochondrial proteins are encoded by nuclear genes and depend on specific targeting signals that direct them from the cytosol, where they are synthesized, into the proper mitochondrial sub-compartments. Mitochondrial DNA (mtDNA) encodes only ~1% of mitochondrial proteins, but 20–25% of the mitochondrial proteome maintains and regulates the mtDNA. The human mtDNA encodes 13 proteins, all of them core parts of mitochondria whose mutation can be associated with higher ROS levels [34]. Many viruses therefore modulate mitochondrial activity, just as they modulate other cellular phenotypes. KSHV infection leads to increased mtDNA, and mitochondrial translation is essential for KSHV latent infection [35]. TBRG4 is a nuclear DNA-encoded protein that is localized in the mitochondria of KSHV- and EBV- infected cells. Depletion of TBRG4 leads to mitochondrial stress by decreasing the efficiency of oxidative phosphorylation. TBRG4 can bind to the majority of heavy strand (guanine rich) mitochondrial RNAs (mtRNA) and is involved in regulating the stability of these mitochondrial transcripts [36]. Knockdown of TBRG4 did not significantly affect the expression level of mtRNAs in KSHV-infected cells, but TBRG4 depletion did significantly alter the expression level of mtDNA-encoded proteins MTND5, MTND6, MDCO2, and MTATP6. Thus, TBRG4-mediated dysregulation of mitochondrial respiration may lead to an increase in ROS byproducts, which can trigger the switch from viral latency to lytic reactivation.

The presence of latent virus in EBV- and KSHV-associated cancers provides a potential target for cancer therapy. Since currently available antiviral drugs target lytic replication but are ineffective against the virus in the latent state, drugs that reactivate the virus have been investigated as therapies for virus-positive neoplasia [37]. Our findings suggest that inhibition of TBRG4 triggers KSHV and EBV lytic reactivation from latency and may be useful in designing novel therapeutic strategies for treating gammaherpesvirus-associated malignancies.

## Materials and methods

### Cell culture

iSLK.219 cells (a kind gift from Dr. Don Ganem) were maintained in DMEM medium (Corning) containing 10% tetracycline (Tet)-free FBS (Sigma), 1% Pen-Strep (Corning), 10 μg/ml puromycin (Corning), 250 μg/ml Geneticin (Corning), and 400 μg/ml hygromycin B (Corning). KSHV-293 cells (a kind gift from J.Vieira) were cultured in DMEM media containing 10% FBS, 1% PS, and 1 μg/ml puromycin. BCBL-1 cells were grown in RPMI 1640 (Corning) medium containing 10% FBS, 1% PS, 1% L-glutamine, 1% sodium bicarbonate and 0.05 mM β-mercaptoethanol. AGS-EBV were grown in F-12 media (Gibco) containing 10% FBS, 1% PS, and 500 μg/ml G418. Akata-BX1 cells were maintained in RPMI 1640 containing 10% FBS, 1% PS and 0.5 mg/ml Geneticin. HEK293FT and HEK293T cells were cultured in DMEM containing 10% FBS and 1% PS. All cells were maintained at 37°C in a 5% CO_2_ laboratory incubator.

### siRNA, viral reactivation, and fluorescence microscopy

iSLK.219 and KSHV-293 cells were transfected with 80 nM TBRG4 siRNA (L-010558-00-0050) (Dharmacon) or control non-targeting (NS) pool using Lipofectamine RNAiMax (Invitrogen). At 48 h post-transfection, iSLK219 cells were replenished with complete DMEM medium containing 0.2 μg/ml Dox. After 72 h post-transfection, KSHV-293 cells were replenished with complete medium including 25 ng/ml TPA and 1mM NaB. Cells were collected at the indicated time for later analysis. GFP and RFP images were taken with a Leica DMi8 microscope with the same exposure time setting. The RFP/GFP fluorescence intensity of images was analyzed with LAS X software or Clariostar plate reader.

### shRNA and KSHV reactivation in BCBL1 cells

The Non-target shRNA control and two shRNAs target TBRG4 (TRCN0000232665 and TRCN0000157762) in the pLKO.5-puro backgrounds were purchased from Sigma Aldrich. Lentivirus for each of the shRNA was produced with the ViraPower Lentiviral Expression System (Invitrogen) in HET293FT cells according to the manufacturer’s instruction. The supernatant containing viral particles was harvested and used to infect BCBL1 with 4 μg/ml polybrene. Infected cells were selected with 1 μg/ml puromycin for 2 d, then the media was changed to complete RPMI 1640 medium without puromycin. After 4 d, infected BCBL1 cells were treated with 25 ng/ml TPA for 16 h and then collected for later analysis.

### Quantitative real-time PCR and qPCR array

Total RNA was extracted with a RNeasy Plus Mini Kit (QIAGEN), and cDNA synthesis was performed using the SensiFAST cDNA Synthesis Kit (Bioline). Real-time qPCR was performed with Sensi-Fast SYBR (Bioline). The housekeeping gene ACTB was used for normalization. The primer sequences are listed in S3 Table. iSLK.219 and KSHV-293 cells were transfected with NS and TBRG4 siRNA and then induced KSHV lytic reactivation for viral qPCR array. Total RNA was extracted with the RNeasy Plus Mini Kit, then poly A^+^ mRNA was purified with the Oligotex mRNA Mini kit (QIAGEN), and cDNA was synthesized using the SensiFAST cDNA synthesis Kit. qPCR was performed with Roche LightCycler 480 and the raw data were shown in S1 Table and S2 Table. A detailed protocol is available at http://www.med.unc.edu/vironomics/protocols. A heat map was generated using the Partek Flow Genomic Analysis software (https://www.partek.com/partek-flow/).

### Immunoblotting

Whole cell lysates were prepared in SDS-sample buffer (60mM Tris.HCl pH6.8, 2% SDS, 10% glycerol, 5% β-mercaptoethanol, 0.01% bromophenol blue), separated by SDS-PAGE, and then transferred to nitrocellulose membranes (GE Healthcare). The membrane was blocked in PBS-T (PBS with 0.1% Tween20) with 5% nonfat milk (Apex), then incubated with the indicated primary antibody at 4°C overnight. Antibodies were obtained from the following source: TBRG4 (PA5-54236), ND5 (66613-1-1G), ND6 (PA5-103954), ATP6 (55313-1-AP), and KSHV ORF45 (MA5-14769) were purchased from Thermo Fisher; Phospho MAPK (4376) and MAPK (4695) were obtained from Cell Signaling; EA-R (sc-56979), Zta (sc-53904), and KSHV K8alpha (sc-57889) were purchased from Santa Cruz; EA-D (EBV-018-48180) was from Capricorn; MT-CO2 (A305-318A) was from Bethyl laboratories; The vIL-6 antibody was purified from the supernatant of v6m 12.1.1 hybridomas (ATCC) using magnetic Protein A/G beads (Thermo Fisher).

### Kaposin mRNA A-to-I editing by Sanger sequencing

iSLK.219 cells were transfected with NS or TBRG4 siRNA for 72 h, and the media was changed with 0.2 μg/ml Dox for 0 or 24 hours. Total RNA was extracted, and cDNA synthesis was performed as described above. Gene-specific amplification was done with Q5 High-Fidelity DNA polymerase (NEB) following the manufacturer’s guidelines. The primer sequences used for amplifying Kaposin are Kaposin R: 5’-GGTGTTTGTGGCAGTTCATG-3’ and F: 5’- AACTCGTGTCCTGAATGCTAC-3’. PCR products were purified with ExoSAP-IT (Thermo Scientific) and then sequenced by Eurofins Genomics. The frequency of A-to-G editing was estimated based on Sanger sequencing with EditR [38].

### Immunofluorescence

Live cells (KSHV-HUVEC, iSLK, and AGS-EBV) were treated with 100 nM MitoTracker (Life Technologles, M22426) and maintained at 37°C in a 5% CO_2_ laboratory incubator. After 25 min, cells were fixed with 4% formaldehyde in PBS at room temperature for 10 min and permeabilized with 0.1% BSA in PBS for 30 min at room temperature. Primary antibody TBRG4 was diluted (1:2000) in PBS with 0.1% Triton-X and 1% BSA and incubated with fixed cells overnight at 4°C. A fluorescently labeled secondary antibody was used to reveal TBRG4 protein. Images were collected using a Leica DMi8.

### Plasmids transfection

A full-length TBRG4 ORF expression vector was purchased from GenScript. The mutant plasmids of TBRG4-ΔMLS and TBRG4-ΔRAP were constructed by deleting amino acids 2–37 in N-terminal and amino acids 555–625 in the C-terminal from full-length TBRG4, respectively. The plasmids were transfected into iSLK.219 cell with using Lipofectamine 2000 (Invitrogen) and then induced KSHV lytic reactivation by adding doxycycline.

### H_2_O_2_ assay

iSLK.219 and AGS-EBV cells were transfected with NS and TBRG4 siRNA, and then viral lytic reactivation was induced with Dox and TPA, respectively. The production of H_2_O_2_ was measured by using a ROS-Glo H_2_O_2_ kit (Promega) according to the manufacturer’s protocol. The assay is based on a luminescent signal generated by a chemical reactivation of H_2_O_2_ and its substrate. The luminescence was measured using a microplate reader. The fold change was calculated and normalized to NS-treated cells at 0 h post reactivation.

### Mito stress test

iSLK.219 cells were transfected with NS and TBRG4 siRNA for 96 h, then seeded in XF24 microplates at a density of 40000 cells/well and maintained at 37°C in a 5% CO_2_ incubator for 16 h. Cells were replenished with Seahorse XF DMEM medium containing 10 mM glucose, 2 mM L-glutamine, and 1 mM sodium pyruvate. The oxygen consumption rates (OCRs) were measured using the Seahorse SXF24 Extracellular Flux Analyzer (Agilent) following the standard protocol. The compounds used included 1.5 μM oligomycin, 0.5 μM FCCP, and 1 μM rotenone and antimycin A.

### Statistical analysis

Statistical significance of differences in mRNA expression level, H_2_O_2_ concentration and viral genome copies were determined using Student’s t-test (two-tailed). For all tests, a P-value of <0.05 was considered statistically significant. * indicates P<0.05.

## Supporting information

S1 Fig Knockdown of TBRG4 increases KSHV lytic reactivation. iSLK.219 cells were transfected with NS and TBRG4 siRNA for 48 h and then treated with Dox (0.2 μg/mL). **(A)** GFP- and RFP-positive cells were imaged 0, 36, and 48 hours post-Dox treatment. **(B)** GFP and RFP intensities were monitored by Leica DMi8, and the RFP/GFP ratio was calculated at the indicated times. **(C)** RNA was extracted from cells and the mRNA expression of TBRG4, FASTK, FASTKD1, FASTKD2, FASTKD3, and FASTKD5 was measured by real-time PCR. (**D**) Uninfected iSLK cells were transfected with NS and TBRG4 siRNA, and the cell viability was determined at the indicated timepoints post-transfection by using a MTT assay. **(E-F)** RNA extracted from KSHV uninfected iSLK cells and infected iSLK.219 cells. The fold induction of IFNβ mRNA expression level was measured by real-time PCR. The data shown are representative of two independent experiments. Data are presented as mean ±SD, P<0.05 by Student’s t-test.
(TIF)

S2 Fig Increased editing of KSHV transcript in TBRG4-depleted cells. **(A)** A-to-G editing of KSHV Kaposin transcripts in iSLK.219 cells. Sanger sequencing of Kaposin cDNA from NS and TBRG4 siRNA-transfected cells at 0 h and 24 h Dox treatment. The frequency of base editing was estimated by Sanger sequencing. **(B)** Schematic representation of A-to-G RNA-editing hairpin loop luciferase reporter. The Stop codon (UAG) leads to no Renilla luciferase expression (top panel), whereas A-to-G editing within the stop codon generates the Trp codon (UGG) and leads to increased expression of Renilla luciferase (bottom panel). **(C)** HEK293T cells were transfected with A-to-G editing reporter luciferase and various plasmids (pcDNA3.1, TBRG4-Flag, or ADAR1-Myc). Luciferase activity was measured 24 h posttransfection in the cell lysates. A plasmid where the edited stop codon (UAG) was mutated to a Trp codon (UGG) was transfected as an A-to-G editing reporter positive control. Western blot was performed using FLAG or Myc antibody accordingly. (**D**) RNA extracted from NS and siTBRG4 siRNA-transfected cells at 0 h and 36 h following Dox-induced lytic reactivation. The Kaposin mRNA expression was measured by real-time PCR. The data shown are representative of two independent experiments. Data are presented as mean ±SD. *, P<0.05 by student’s t test.
(TIF)

S3 Fig The MLS and RAP domains of TBRG4 are required for its inhibition of KSHV lytic reactivation. iSLK.219 cells were transfected with empty vector, plasmids expressing full-length TBRG4, TBRG4-ΔMLS, or TBRG4-ΔRAP, and then treated with Dox (0.2 μg/ml) for 24 h. (**A**) Schematic of TBRG4 and its deletion mutants. (**B**) Cell lysates were harvested following Dox induction and Western blots were performed with indicated antibodies. (**C**-**F**) RNA was extracted from the cells and the mRNA expression level of TBRG4 and KSHV viral genes were measured by real-time PCR.
(TIF)

S4 Fig Knockdown of TBRG4 increased ERK1/2 phosphorylation and an ERK inhibitor affects KSHV lytic reactivation in TBRG4-depleted cells. (**A**) iSLK cells were treated with MitoTracker to visualize the mitochondria (red), then fixed and stained with TBRG4 antibody (green). Images were collected using a Leica DMi8. (**B**) iSLK.219 cells were transfected with NS and TBRG4 siRNA for 48 hours. The RNA was extracted from the cells and the expression level of mitochondrial mRNA was measured by real-time PCR and normalized to β-actin mRNA. **(C)** HEK293T cells were transfected with NS and TBRG4 siRNA for 48 h followed by transfection with KSHV ORF50 promoter-luciferase reporter plasmids and TK-driven control renilla pGL4.73 plasmids. The luciferase activity was measured in the cell lysates 24 h post-transfection of these plasmids. (**D**-**I**) iSLK.219 cells were treated with NS siRNA, siTBRG4 or siTBRG4 along with 10 μM U0126 ERK1/2 inhibitor, and then treated with Dox for 48 h. The mRNA expression of TBRG4 and KSHV viral genes was measured by real-time PCR. Western blots were performed with the indicated antibodies.
(TIF)

S5 Fig Depletion of TBRG4 induces EBV lytic reactivation in Akata-BX1 cells. Akata-BX1 cells were infected with lentivirus expressing an NTC shRNA or a shRNA targeting TBRG4 and then treated with human IgG for 24 h to reactivate EBV. (**A**-**E**) RNA was extracted from the cells and the mRNA expression of TBRG4, LMP1, BMRF1, BALF2, and BLLF1 was measured by real-time PCR. (**F**) Western blots were performed with the indicated antibodies.
(TIF)

S6 Fig NAC treatment inhibits EBV lytic reactivation in TBRG4-depleted cells. AGS-EBV cells were transfected with NS, siTBRG4, or siTBRG4 along with NAC for 48 h and then treated with TPA for 24 h. (**A**) The level of H_2_O_2_ was measured with ROS-Glo H2O2 assay and is displayed as fold induction. (**B**-**F**) The mRNA expression of TBRG4 and EBV viral genes was measured by real-time PCR. (**G**) Western blots were performed with the indicated antibodies.
(TIF)

S1 Table KSHV PCR array in iSLK.219 cells. (PDF)

S2 Table KSHV PCR array in KSHV-293 cells. (PDF)

S3 Table Primer sequences. (PDF)

## References

[1] Cesarman E, Chang Y, Moore PS, Said JW, Knowles DM. Kaposis Sarcoma-Associated Herpesvirus-Like DNA-Sequences in Aids-Related Body-Cavity-Based Lymphomas. New Engl J Med. 1995;332(18):1186–91. WOS:A1995QV41500002. doi: 10.1056/NEJM199505043321802 7700311

[2] Chang Y, Cesarman E, Pessin MS, Lee F, Culpepper J, Knowles DM, et al. Identification of Herpesvirus-Like DNA-Sequences in Aids-Associated Kaposis-Sarcoma. Science. 1994;266(5192):1865–9. WOS:A1994PX38300036. doi: 10.1126/science.7997879 7997879

[3] Soulier J, Grollet L, Oksenhendler E, Cacoub P, Cazalshatem D, Babinet P, et al. Kaposis Sarcoma-Associated Herpesvirus-Like DNA-Sequences in Multicentric Castlemans Disease. Blood. 1995;86(4):1276–80. WOS:A1995RN46700005. 7632932

[4] Epstein MA, Achong BG, Barr YM. Virus Particles in Cultured Lymphoblasts from Burkitt’s Lymphoma. Lancet. 1964;1(7335):702–3. Epub 1964/03/28. doi: 10.1016/s0140-6736(64)91524-7 . 14107961

[5] Henle G, Henle W. Immunofluorescence in cells derived from Burkitt’s lymphoma. J Bacteriol. 1966;91(3):1248–56. Epub 1966/03/01. doi: 10.1128/jb.91.3.1248-1256.1966 ; PMCID: PMC316020. 4160230 PMC316020

[6] Damania B, Kenney SC, Raab-Traub N. Epstein-Barr virus: Biology and clinical disease. Cell. 2022 Sep 29;185(20):3652–3670. 36113467 10.1016/j.cell.2022.08.026 PMC9529843

[7] Miller G, Heston L, Grogan E, Gradoville L, Rigsby M, Sun R, et al. Selective switch between latency and lytic replication of Kaposi’s sarcoma herpesvirus and Epstein-Barr virus in dually infected body cavity lymphoma cells. J Virol. 1997;71(1):314–24. WOS:A1997VX29200037. doi: 10.1128/JVI.71.1.314-324.1997 8985352 PMC191053

[8] Kenney SC, Mertz JE. Regulation of the latent-lytic switch in Epstein-Barr virus. Semin Cancer Biol. 2014;26:60–8. Epub 2014/01/25. doi: 10.1016/j.semcancer.2014.01.002 ; PMCID: PMC4048781. 24457012 PMC4048781

[9] Broussard G, Damania B. Regulation of KSHV Latency and Lytic Reactivation. Viruses-Basel. 2020;12(9). WOS:000580225000001. doi: 10.3390/v12091034 32957532 PMC7551196

[10] Lange PT, White MC, Damania B. Activation and Evasion of Innate Immunity by Gammaherpesviruses. J Mol Biol. 2022;434(6):167214. Epub 2021/08/27. doi: 10.1016/j.jmb.2021.167214 ; PMCID: PMC8863980. 34437888 PMC8863980

[11] Li H, Liu S, Hu J, Luo X, Li N, A MB, et al. Epstein-Barr virus lytic reactivation regulation and its pathogenic role in carcinogenesis. Int J Biol Sci. 2016;12(11):1309–18. Epub 2016/11/24. doi: 10.7150/ijbs.16564 ; PMCID: PMC5118777. 27877083 PMC5118777

[12] Purushothaman P, Uppal T, Verma SC. Molecular Biology of KSHV Lytic Reactivation. Viruses-Basel. 2015;7(1):116–53. WOS:000348401600006. doi: 10.3390/v7010116 25594835 PMC4306831

[13] Mallery SR, Pei P, Landwehr DJ, Clark CM, Bradburn JE, Ness GM, et al. Implications for oxidative and nitrative stress in the pathogenesis of AIDS-related Kaposi’s sarcoma. Carcinogenesis. 2004;25(4):597–603. WOS:000220485700016. doi: 10.1093/carcin/bgh042 14656937 PMC2405907

[14] Bottero V, Chakraborty S, Chandran B. Reactive Oxygen Species Are Induced by Kaposi’s Sarcoma-Associated Herpesvirus Early during Primary Infection of Endothelial Cells To Promote Virus Entry. J Virol. 2013;87(3):1733–49. WOS:000313558100039. doi: 10.1128/JVI.02958-12 23175375 PMC3554172

[15] Li XD, Feng JY, Sun R. Oxidative Stress Induces Reactivation of Kaposi’s Sarcoma-Associated Herpesvirus and Death of Primary Effusion Lymphoma Cells. J Virol. 2011;85(2):715–24. WOS:000285554300007. doi: 10.1128/JVI.01742-10 21068240 PMC3020037

[16] Ye FC, Zhou FC, Bedolla RG, Jones T, Lei XF, Kang T, et al. Reactive Oxygen Species Hydrogen Peroxide Mediates Kaposi’s Sarcoma-Associated Herpesvirus Reactivation from Latency. Plos Pathog. 2011;7(5). WOS:000291014000038. doi: 10.1371/journal.ppat.1002054 21625536 PMC3098240

[17] Lassoued S, Ben Ameur R, Ayadi W, Gargouri B, Ben Mansour R, Attia H. Epstein-Barr virus induces an oxidative stress during the early stages of infection in B lymphocytes, epithelial, and lymphoblastoid cell lines. Mol Cell Biochem. 2008;313(1–2):179–86. Epub 2008/04/17. doi: 10.1007/s11010-008-9755-z . 18414998

[18] Gargouri B, Nasr R, ben Mansour R, Lassoued S, Mseddi M, Attia H, et al. Reactive oxygen species production and antioxidant enzyme expression after Epstein-Barr virus lytic cycle induction in Raji cell line. Biol Trace Elem Res. 2011;144(1–3):1449–57. Epub 2011/07/28. doi: 10.1007/s12011-011-9135-5 . 21792596

[19] Jourdain AA, Popow J, de la Fuente MA, Martinou JC, Anderson P, Simarro M. The FASTK family of proteins: emerging regulators of mitochondrial RNA biology. Nucleic Acids Res. 2017;45(19):10941–7. Epub 2017/10/17. doi: 10.1093/nar/gkx772 ; PMCID: PMC5737537. 29036396 PMC5737537

[20] Ohkubo A, Van Haute L, Rudler DL, Stentenbach M, Steiner FA, Rackham O, et al. The FASTK family proteins fine-tune mitochondrial RNA processing. PLoS Genet. 2021;17(11):e1009873. Epub 2021/11/09. doi: 10.1371/journal.pgen.1009873 ; PMCID: PMC8601606. 34748562 PMC8601606

[21] Huang F, Liao F, Ma G, Hu Y, Zhang C, Xu P, et al. TBRG4 Knockdown Suppresses Proliferation and Growth of Human Osteosarcoma Cell Lines MG63 Through PI3K/Akt Pathway. Onco Targets Ther. 2020;13:7271–81. Epub 2020/08/18. doi: 10.2147/OTT.S249477 ; PMCID: PMC7394601. 32801755 PMC7394601

[22] Wang J, Luo Q, Liu M, Zhang C, Jia Y, Tong R, et al. TBRG4 silencing promotes progression of squamous cell carcinoma via regulation of CAV-1 expression and ROS formation. Cell Mol Biol (Noisy-le-grand). 2020;66(2):157–64. Epub 2020/05/18. . 32415943

[23] Zhang H, Ni G, Damania B. ADAR1 Facilitates KSHV Lytic Reactivation by Modulating the RLR-Dependent Signaling Pathway. Cell Rep. 2020;31(4):107564. Epub 2020/04/30. doi: 10.1016/j.celrep.2020.107564 ; PMCID: PMC7319254. 32348766 PMC7319254

[24] Cohen A, Brodie C, Sarid R. An essential role of ERK signalling in TPA-induced reactivation of Kaposi’s sarcoma-associated herpesvirus. J Gen Virol. 2006;87:795–802. WOS:000236446400007. doi: 10.1099/vir.0.81619-0 16528027

[25] Huang SY, Fang CY, Wu CC, Tsai CH, Lin SF, Chen JY. Reactive Oxygen Species Mediate Epstein-Barr Virus Reactivation by N-Methyl-N’-Nitro-N-Nitrosoguanidine. Plos One. 2013;8(12). WOS:000328745100192. doi: 10.1371/journal.pone.0084919 24376853 PMC3869928

[26] Hu JM, Li YS, Li HD, Shi F, Xie LL, Zhao L, et al. Targeting Epstein-Barr virus oncoprotein LMP1-mediated high oxidative stress suppresses EBV lytic reactivation and sensitizes tumors to radiation therapy. Theranostics. 2020;10(26):11921–37. WOS:000592299200006. doi: 10.7150/thno.46006 33204320 PMC7667690

[27] Chang PC, Fitzgerald LD, Van Geelen A, Izumiya Y, Ellison TJ, Wang DH, et al. Kruppel-Associated Box Domain-Associated Protein-1 as a Latency Regulator for Kaposi’s Sarcoma-Associated Herpesvirus and Its Modulation by the Viral Protein Kinase. Cancer Res. 2009;69(14):5681–9. WOS:000268360300012. doi: 10.1158/0008-5472.CAN-08-4570 19584288 PMC2731626

[28] Gwack Y, Nakamura H, Lee SH, Souvlis J, Yustein JT, Gygi S, et al. Poly(ADP-ribose) polymerase 1 and Ste20-like kinase hKFC act as transcriptional repressors for gamma-2 herpesvirus lytic replication. Mol Cell Biol. 2003;23(22):8282–94. WOS:000186354800031. doi: 10.1128/MCB.23.22.8282-8294.2003 14585985 PMC262387

[29] Ma Z, Hopcraft SE, Yang F, Petrucelli A, Guo H, Ting JP, et al. NLRX1 negatively modulates type I IFN to facilitate KSHV reactivation from latency. Plos Pathog. 2017;13(5):e1006350. Epub 2017/05/02. doi: 10.1371/journal.ppat.1006350 ; PMCID: PMC5426799. 28459883 PMC5426799

[30] West JA, Wicks M, Gregory SM, Chugh P, Jacobs SR, Zhang Z, et al. An important role for mitochondrial antiviral signaling protein in the Kaposi’s sarcoma-associated herpesvirus life cycle. J Virol. 2014;88(10):5778–87. Epub 2014/03/14. doi: 10.1128/JVI.03226-13 ; PMCID: PMC4019080. 24623417 PMC4019080

[31] Cerimele F, Battle T, Lynch R, Frank DA, Murad E, Cohen C, et al. Reactive oxygen signaling and MAPK activation distinguish Epstein-Barr virus (EBV)-positive versus EBV-negative Burkitt’s lymphoma. P Natl Acad Sci USA. 2005;102(1):175–9. WOS:000226216400032. doi: 10.1073/pnas.0408381102 15611471 PMC544042

[32] Bubman D, Csernus B, Guasparri I, Cesarman E. Reactive Oxygen Species (ROS) are induced by vFLIP and regulate NF-kB activation in KSHV-infected primary effusion lymphoma cells. Blood. 2005;106(11):291a–a. WOS:000233426001461.

[33] Hu J, Li Y, Li H, Shi F, Xie L, Zhao L, et al. Targeting Epstein-Barr virus oncoprotein LMP1-mediated high oxidative stress suppresses EBV lytic reactivation and sensitizes tumors to radiation therapy. Theranostics. 2020;10(26):11921–37. Epub 2020/11/19. doi: 10.7150/thno.46006 ; PMCID: PMC7667690. 33204320 PMC7667690

[34] Pfanner N, Warscheid B, Wiedemann N. Mitochondrial proteins: from biogenesis to functional networks. Nat Rev Mol Cell Biol. 2019;20(5):267–84. Epub 2019/01/11. doi: 10.1038/s41580-018-0092-0 ; PMCID: PMC6684368. 30626975 PMC6684368

[35] Holmes DL, Vogt DT, Lagunoff M. A CRISPR-Cas9 screen identifies mitochondrial translation as an essential process in latent KSHV infection of human endothelial cells. Proc Natl Acad Sci U S A. 2020;117(45):28384–92. Epub 2020/10/31. doi: 10.1073/pnas.2011645117 ; PMCID: PMC7668072. 33122441 PMC7668072

[36] Van Nostrand EL, Freese P, Pratt GA, Wang X, Wei X, Xiao R, et al. A large-scale binding and functional map of human RNA-binding proteins. Nature. 2020;583(7818):711–9. Epub 2020/07/31. doi: 10.1038/s41586-020-2077-3 ; PMCID: PMC7410833. 32728246 PMC7410833

[37] Jha HC, Banerjee S, Robertson ES. The Role of Gammaherpesviruses in Cancer Pathogenesis. Pathogens. 2016;5(1). Epub 2016/02/11. doi: 10.3390/pathogens5010018 ; PMCID: PMC4810139. 26861404 PMC4810139

[38] Kluesner MG, Nedveck DA, Lahr WS, Garbe JR, Abrahante JE, Webber BR, et al. EditR: A Method to Quantify Base Editing from Sanger Sequencing. CRISPR J. 2018;1:239–50. Epub 2019/04/26. doi: 10.1089/crispr.2018.0014 ; PMCID: PMC6694769. 31021262 PMC6694769

