# Supplementary figures and images for: Mitochondrial protein, TBRG4, modulates KSHV and EBV reactivation from latency
Source: PLoS Pathog. 2022 Nov 23;18(11):e1010990. doi: 10.1371/journal.ppat.1010990 (PMC9683600; doi:10.1371/journal.ppat.1010990)

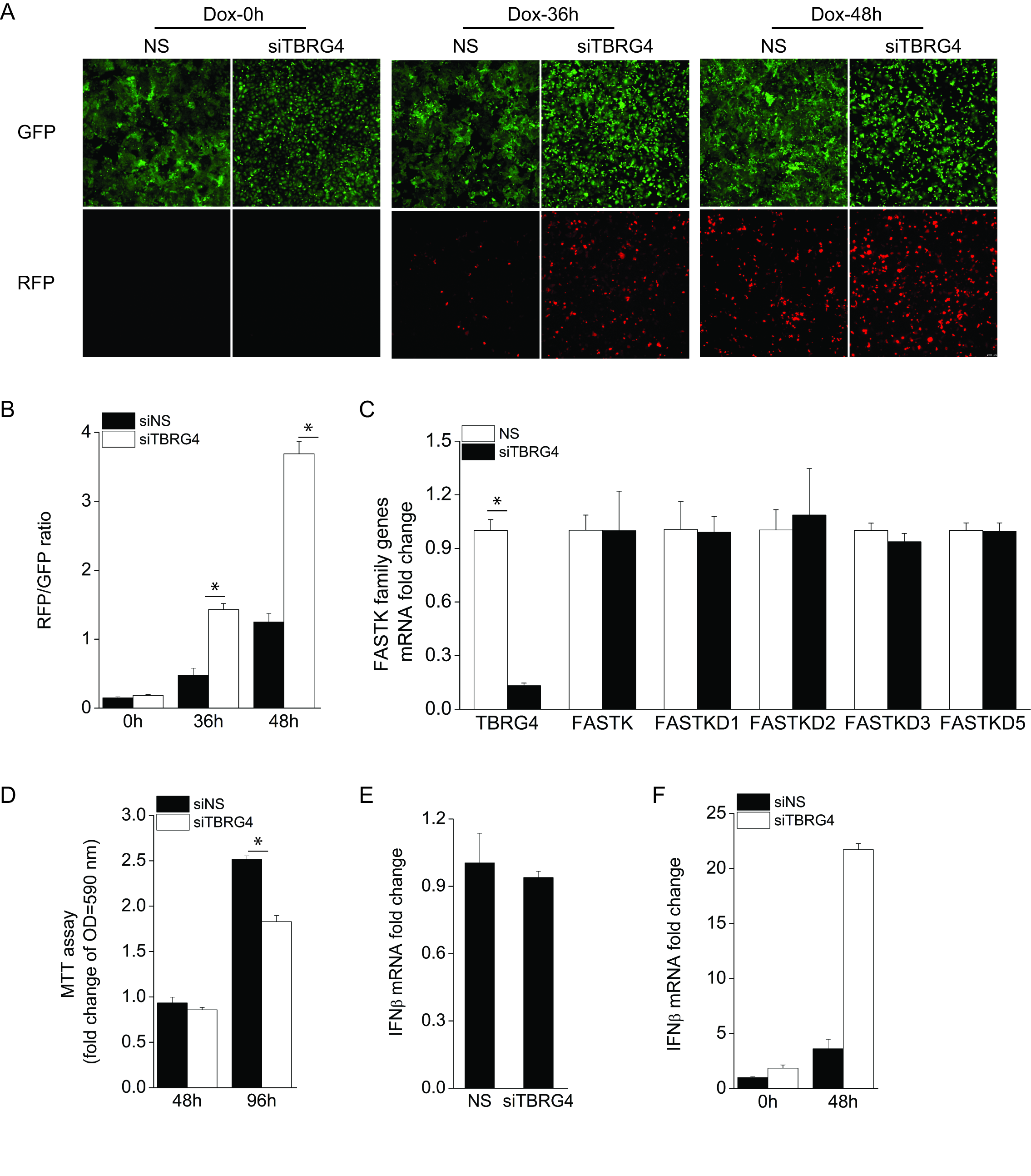

Supplement: S1 Fig — iSLK.219 cells were transfected with NS and TBRG4 siRNA for 48 h and then treated with Dox (0.2 μg/mL). (A) GFP- and RFP-positive cells were imaged 0, 36, and 48 hours post-Dox treatment. (B) GFP and RFP intensities were monitored by Leica DMi8, and the RFP/GFP ratio was calculated at the indicated times. (C) RNA was extracted from cells and the mRNA expression of TBRG4, FASTK, FASTKD1, FASTKD2, FASTKD3, and FASTKD5 was measured by real-time PCR. (D) Uninfected iSLK cells were transfected with NS and TBRG4 siRNA, and the cell viability was determined at the indicated timepoints post-transfection by using a MTT assay. (E-F) RNA extracted from KSHV uninfected iSLK cells and infected iSLK.219 cells. The fold induction of IFNβ mRNA expression level was measured by real-time PCR. The data shown are representative of two independent experiments. Data are presented as mean ±SD, P<0.05 by Student’s t-test. (TIF) [file ppat.1010990.s001.tif]

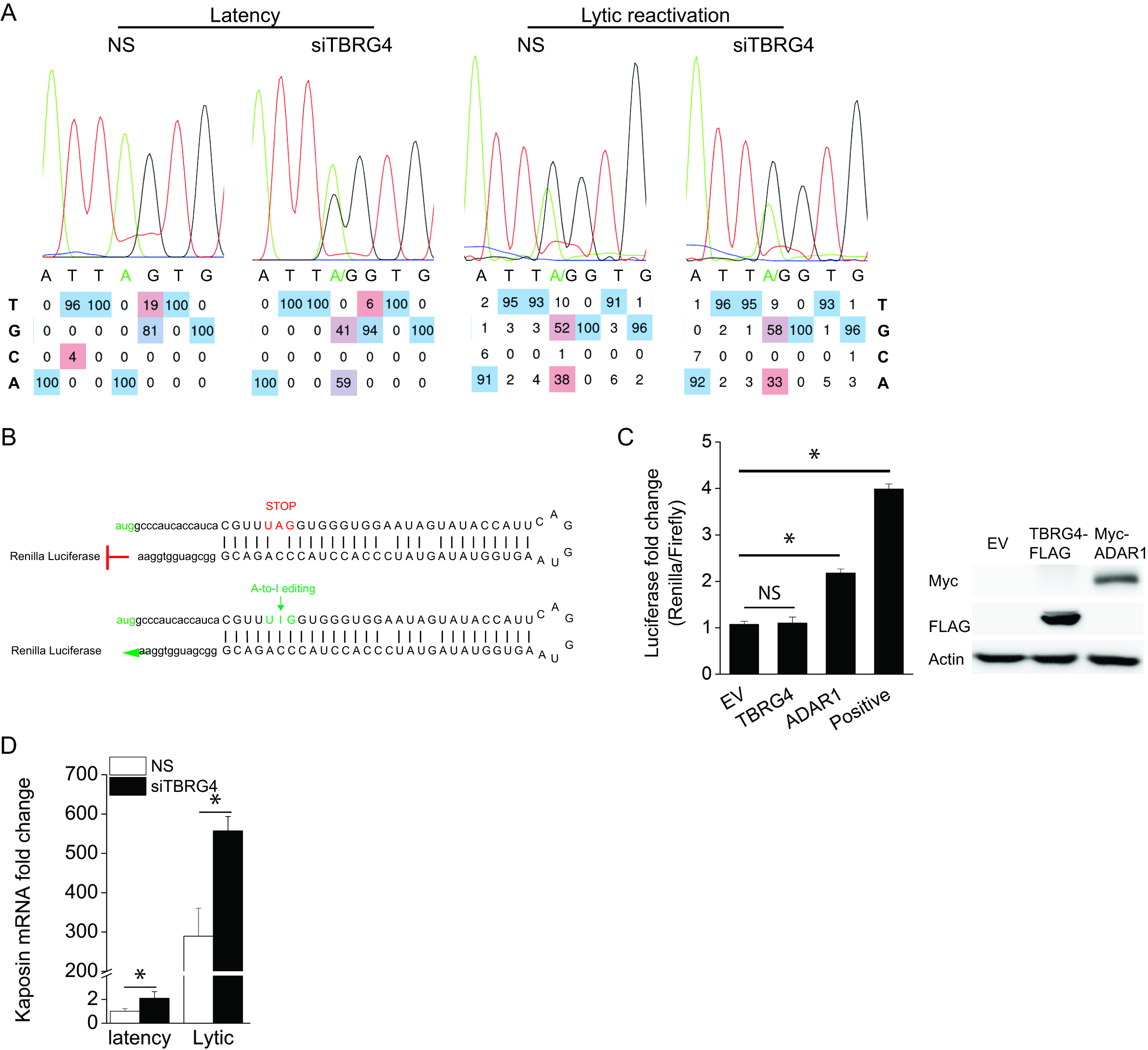

Supplement: S2 Fig — (A) A-to-G editing of KSHV Kaposin transcripts in iSLK.219 cells. Sanger sequencing of Kaposin cDNA from NS and TBRG4 siRNA-transfected cells at 0 h and 24 h Dox treatment. The frequency of base editing was estimated by Sanger sequencing. (B) Schematic representation of A-to-G RNA-editing hairpin loop luciferase reporter. The Stop codon (UAG) leads to no Renilla luciferase expression (top panel), whereas A-to-G editing within the stop codon generates the Trp codon (UGG) and leads to increased expression of Renilla luciferase (bottom panel). (C) HEK293T cells were transfected with A-to-G editing reporter luciferase and various plasmids (pcDNA3.1, TBRG4-Flag, or ADAR1-Myc). Luciferase activity was measured 24 h posttransfection in the cell lysates. A plasmid where the edited stop codon (UAG) was mutated to a Trp codon (UGG) was transfected as an A-to-G editing reporter positive control. Western blot was performed using FLAG or Myc antibody accordingly. (D) RNA extracted from NS and siTBRG4 siRNA-transfected cells at 0 h and 36 h following Dox-induced lytic reactivation. The Kaposin mRNA expression was measured by real-time PCR. The data shown are representative of two independent experiments. Data are presented as mean ±SD. *, P<0.05 by student’s t test. (TIF) [file ppat.1010990.s002.tif]

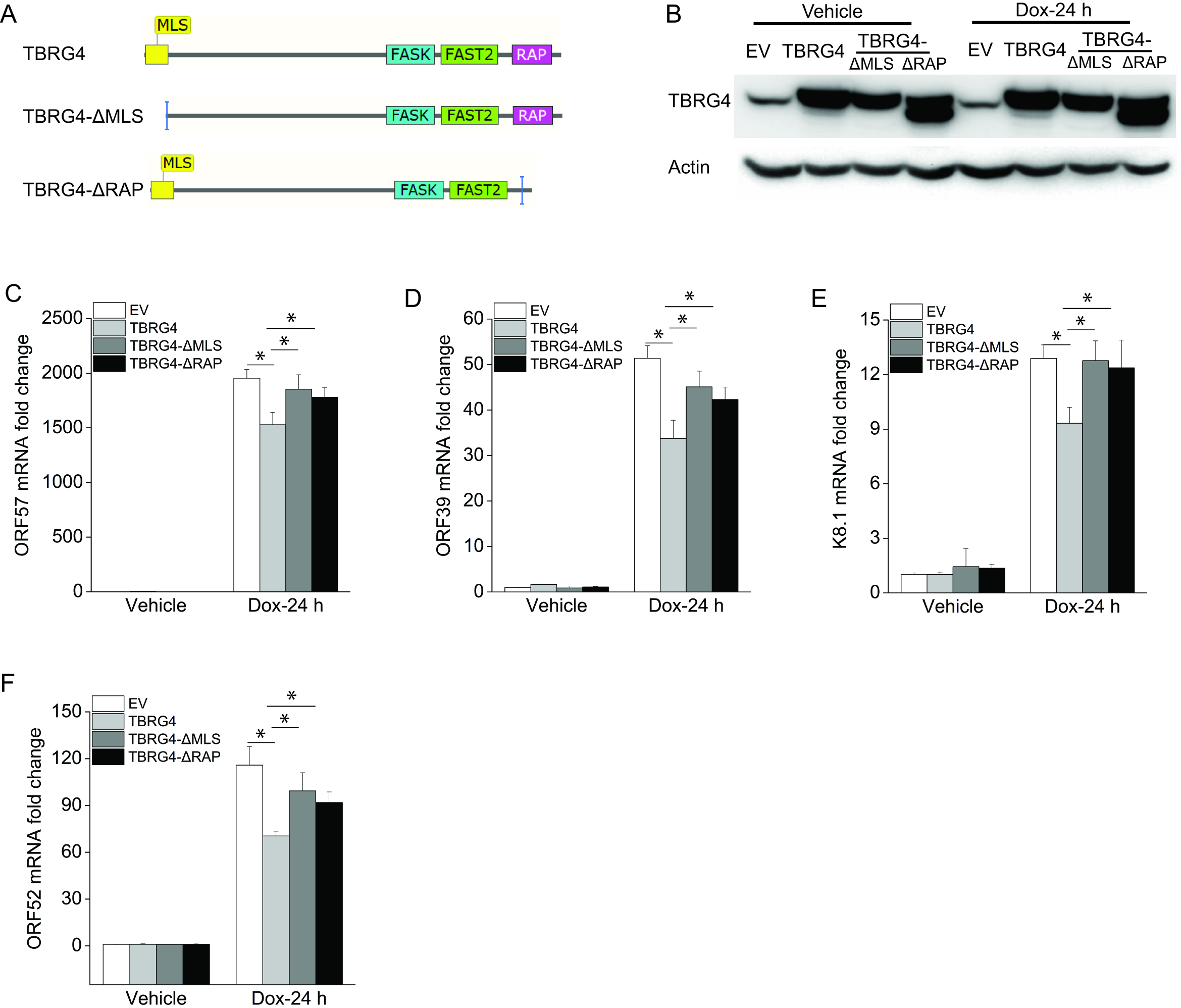

Supplement: S3 Fig — iSLK.219 cells were transfected with empty vector, plasmids expressing full-length TBRG4, TBRG4-ΔMLS, or TBRG4-ΔRAP, and then treated with Dox (0.2 μg/ml) for 24 h. (A) Schematic of TBRG4 and its deletion mutants. (B) Cell lysates were harvested following Dox induction and Western blots were performed with indicated antibodies. (C-F) RNA was extracted from the cells and the mRNA expression level of TBRG4 and KSHV viral genes were measured by real-time PCR. (TIF) [file ppat.1010990.s003.tif]

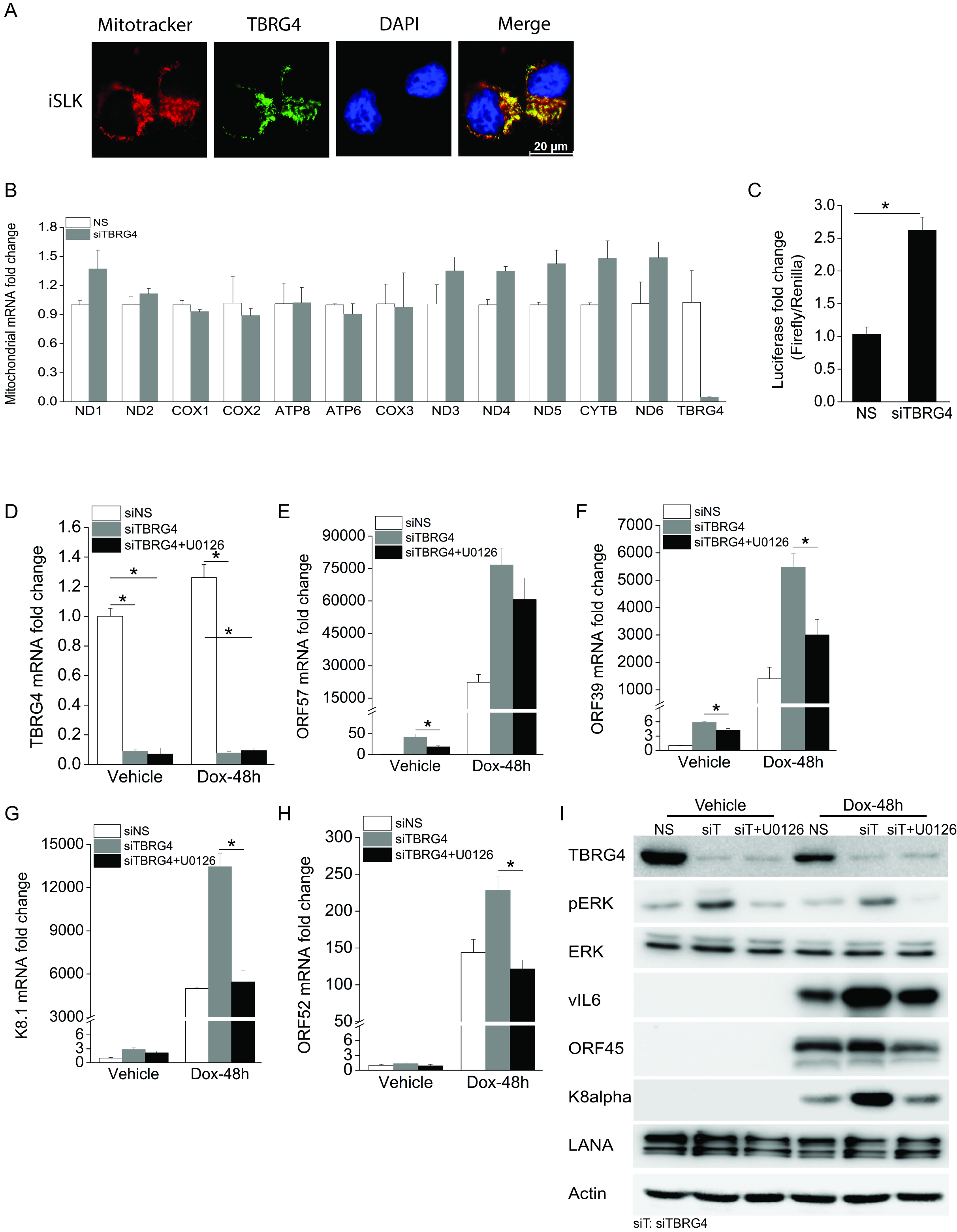

Supplement: S4 Fig — (A) iSLK cells were treated with MitoTracker to visualize the mitochondria (red), then fixed and stained with TBRG4 antibody (green). Images were collected using a Leica DMi8. (B) iSLK.219 cells were transfected with NS and TBRG4 siRNA for 48 hours. The RNA was extracted from the cells and the expression level of mitochondrial mRNA was measured by real-time PCR and normalized to β-actin mRNA. (C) HEK293T cells were transfected with NS and TBRG4 siRNA for 48 h followed by transfection with KSHV ORF50 promoter-luciferase reporter plasmids and TK-driven control renilla pGL4.73 plasmids. The luciferase activity was measured in the cell lysates 24 h post-transfection of these plasmids. (D-I) iSLK.219 cells were treated with NS siRNA, siTBRG4 or siTBRG4 along with 10 μM U0126 ERK1/2 inhibitor, and then treated with Dox for 48 h. The mRNA expression of TBRG4 and KSHV viral genes was measured by real-time PCR. Western blots were performed with the indicated antibodies. (TIF) [file ppat.1010990.s004.tif]

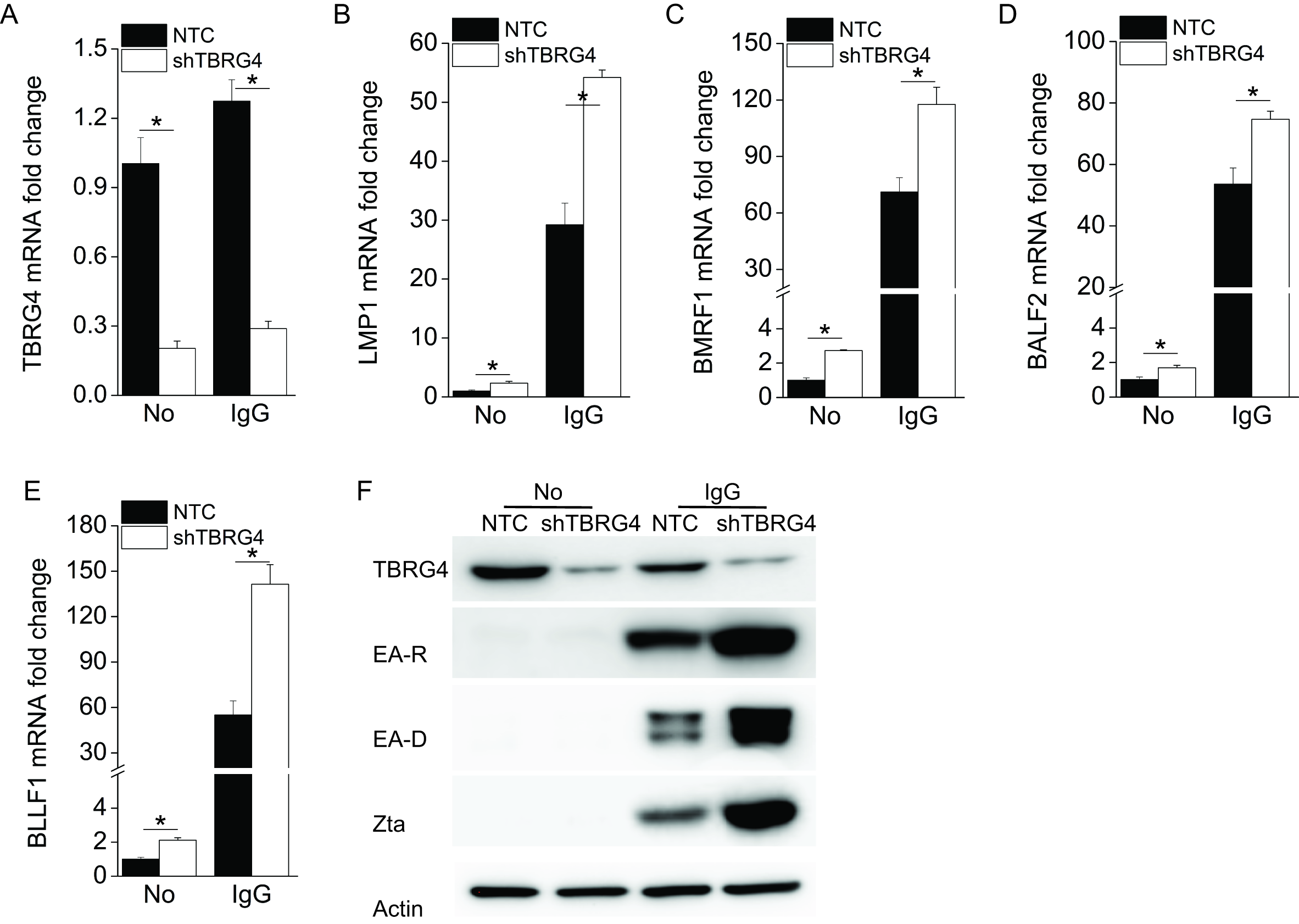

Supplement: S5 Fig — Akata-BX1 cells were infected with lentivirus expressing an NTC shRNA or a shRNA targeting TBRG4 and then treated with human IgG for 24 h to reactivate EBV. (A-E) RNA was extracted from the cells and the mRNA expression of TBRG4, LMP1, BMRF1, BALF2, and BLLF1 was measured by real-time PCR. (F) Western blots were performed with the indicated antibodies. (TIF) [file ppat.1010990.s005.tif]

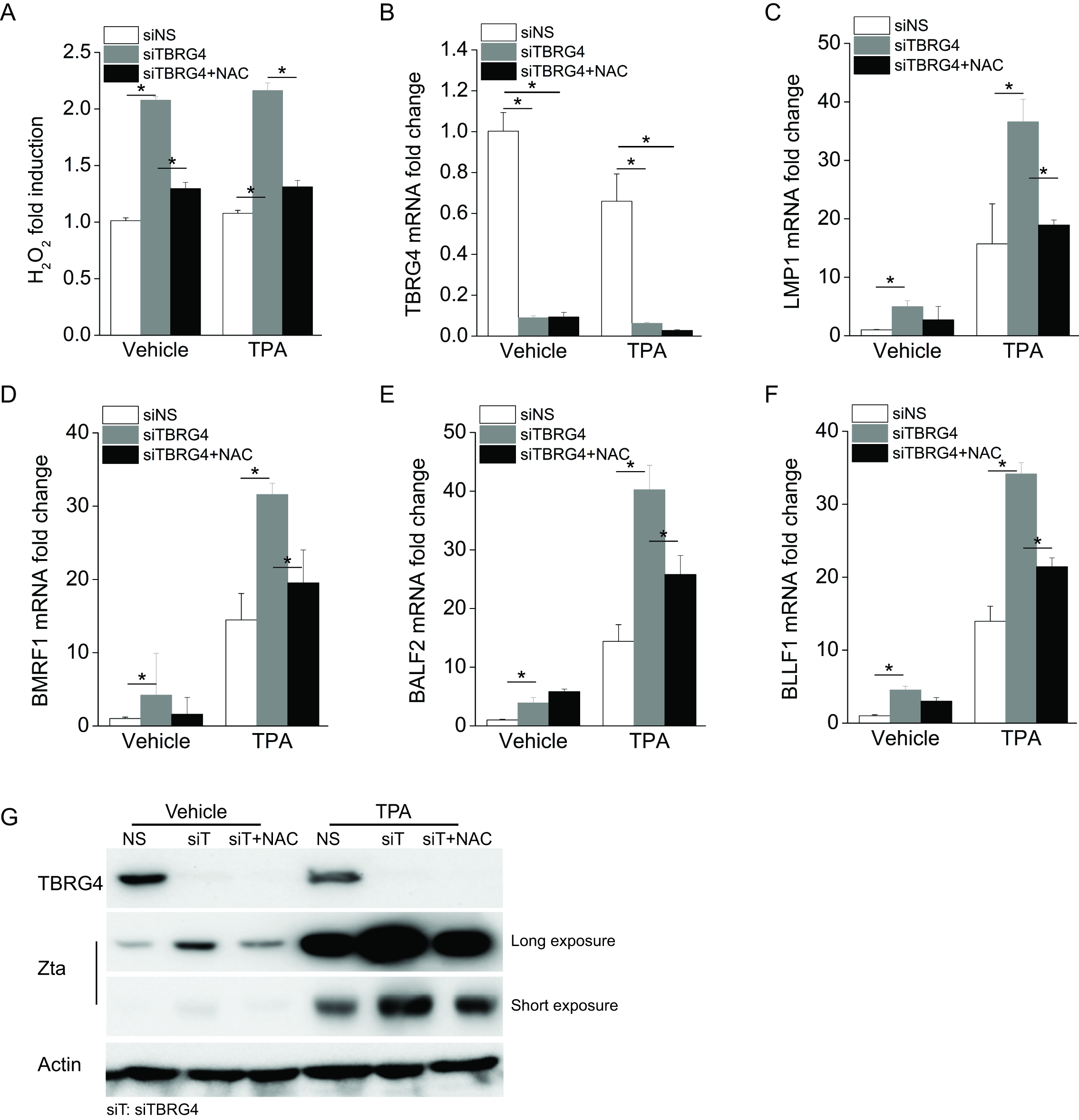

Supplement: S6 Fig — AGS-EBV cells were transfected with NS, siTBRG4, or siTBRG4 along with NAC for 48 h and then treated with TPA for 24 h. (A) The level of H2O2 was measured with ROS-Glo H2O2 assay and is displayed as fold induction. (B-F) The mRNA expression of TBRG4 and EBV viral genes was measured by real-time PCR. (G) Western blots were performed with the indicated antibodies. (TIF) [file ppat.1010990.s006.tif]
