# Supplementary material for: Mitochondrial protein, TBRG4, modulates KSHV and EBV reactivation from latency
Source: PLoS Pathog. 2022 Nov 23;18(11):e1010990. doi: 10.1371/journal.ppat.1010990 (PMC9683600; doi:10.1371/journal.ppat.1010990)
Supplement: S1 Table — (PDF) [file ppat.1010990.s007.pdf]

S1 Table. KSHV PCR array in iSLK.219 cells

	Delta Ct											
Target Name	NS Dox0h-R1	NS Dox0h-R2	TBRG4 Dox0h-R1	TBRG4 Dox0h-R2	NS Dox24 h-R1	NS Dox24 h-R2	TBRG4 Dox24 h-R1	TBRG4 Dox24 h-R2	NS Dox48 h-R1	NS Dox48 h-R2	TBRG4 Dox48 h-R1	TBRG4 Dox48 h-R2
ORF9_13975	13.497	13.278	11.767	11.598	6.419	6.207	4.043	3.870	3.391	3.227	0.512	0.697
K1_622	7.777	7.585	7.531	7.197	6.919	6.744	5.627	5.670	5.387	5.398	2.963	3.518
K3_18883	10.514	10.998	9.520	9.106	3.305	2.771	2.220	2.079	0.932	0.791	-0.823	-0.815
K2_17821	3.406	3.280	3.712	3.678	-1.935	-2.181	-3.139	-2.737	-1.910	-1.791	-2.916	-2.459
ORF11_16083	11.097	10.556	8.552	8.152	1.754	1.131	-0.003	-0.213	0.281	0.440	-1.040	-0.992
ORF10_15288	14.465	13.504	11.889	11.672	6.221	5.910	4.320	4.096	3.306	3.162	0.878	1.568
ORF22_38336	15.329	14.787	12.279	11.474	5.583	5.156	4.031	3.954	2.660	2.744	1.243	1.246
ORF34_55680	15.089	14.363	10.207	9.481	5.169	5.001	3.282	3.030	2.475	2.462	0.184	0.386
ORF36_56709	14.096	13.529	9.399	8.705	4.443	4.341	2.454	2.342	1.515	1.602	-0.652	-0.436
ORF16_30403	14.319	13.506	10.405	10.203	5.338	4.972	4.142	3.975	3.300	3.507	2.085	2.225
LANA78	7.577	7.449	8.255	8.524	6.821	6.590	6.691	6.490	6.070	6.203	3.954	3.677
ORF50_74850	9.505	9.527	7.341	6.931	0.500	0.489	-0.869	-1.028	-0.080	0.059	-2.053	-1.985
ORF21_36500	14.945	15.539	12.969	12.806	6.867	6.595	5.315	5.260	4.182	4.186	2.694	3.149
ORF71_VFLIP_122381	7.309	7.215	7.846	8.012	6.099	5.938	5.819	5.785	4.103	4.188	2.115	1.995
ORF73_LANA_124002	8.467	8.223	8.936	9.223	7.398	7.783	7.743	7.780	7.081	7.372	5.017	4.912
LANA78	7.689	7.390	8.193	8.474	6.866	6.624	6.961	6.699	6.013	6.296	3.939	3.679
ORF17_32510	14.766	14.305	12.235	11.935	6.214	5.850	5.447	5.279	3.521	3.389	1.696	1.801
VGPCR_129945	13.698	13.268	12.835	12.866	7.753	7.442	5.653	5.420	3.963	4.291	1.147	0.745
VGPCR_130126	14.128	13.892	13.042	12.749	7.952	7.465	5.831	5.592	4.210	4.318	1.251	0.798
ORF75_130749	11.172	10.881	10.707	10.530	9.433	8.975	8.402	8.224	6.945	6.883	4.214	4.104
K15_131117	10.629	10.460	10.202	9.986	8.647	8.621	8.055	8.019	6.340	6.488	3.853	3.802
ORF65_112376	16.535	16.029	16.988	15.239	9.405	9.110	8.496	7.229	6.842	6.800	4.376	4.581
K3_18883	10.610	10.764	9.299	9.011	3.255	2.834	2.052	2.067	0.861	0.841	-0.786	-0.839
K4_21778	10.966	10.498	8.331	8.015	2.293	2.080	1.325	1.401	-0.040	0.065	-0.907	-1.051
K7_28624	10.839	10.656	9.324	9.201	6.395	6.488	4.383	4.905	4.744	5.018	2.993	2.656
K5_26145	10.660	10.626	10.171	9.793	4.000	3.899	2.899	2.896	1.492	1.551	0.053	0.373
ORF17_31509	11.775	11.796	9.057	8.800	4.519	4.195	3.384	3.068	0.980	1.058	-1.116	-0.854
ORF18_32935	15.441	14.290	11.955	11.811	6.443	6.004	4.932	4.783	3.495	3.613	1.709	1.841
ORF63_103119	17.136	16.858	17.131	16.643	11.322	11.469	10.131	10.259	8.288	8.113	6.025	6.088
ORF64_111231	15.976	16.017	16.891	15.947	10.720	10.910	8.901	8.985	6.787	6.643	4.364	4.055
ORF66_114453	13.027	13.919	13.415	13.682	7.426	7.485	5.871	5.825	5.332	5.413	2.961	3.022
ORF67_113980	14.154	14.699	15.212	14.296	8.673	8.447	7.199	7.025	6.333	6.184	3.902	3.595
ORF68_115939	12.419	12.297	12.856	11.997	5.809	5.610	4.309	4.267	4.110	4.024	1.568	1.888
ORF69_117120	12.510	12.557	12.450	11.785	5.875	5.476	4.309	4.256	3.529	3.519	0.898	0.965
ORF7_8384	16.216	15.865	16.035	16.219	10.941	10.513	8.836	8.801	6.609	6.676	3.686	3.909
ORF70_20979	14.767	14.566	12.024	11.851	3.760	3.837	2.698	2.608	1.476	1.561	-0.159	-0.156
VCYC_123054	7.884	7.680	8.787	8.901	6.856	6.868	6.595	6.516	4.724	4.739	2.975	2.968
ORF8_10765	14.336	13.767	12.099	11.919	7.522	7.386	4.905	4.884	3.823	3.709	0.886	1.535
ORF19_34089	16.817	15.535	13.191	12.763	7.028	6.396	5.190	5.103	3.760	3.740	1.973	2.102
ORF20_34848	18.990	16.798	15.581	15.876	8.746	8.670	7.473	7.389	5.920	5.889	3.962	4.131
ORF23_39786	18.394	15.994	14.081	14.437	9.278	8.955	7.223	7.066	5.903	5.842	3.665	3.408
ORF24_41630	16.533	15.347	15.257	14.012	9.680	9.230	7.366	7.021	6.099	5.998	3.793	3.862

ORF25_45767	15.665	15.522	13.813	13.625	9.385	9.159	6.437	6.402	5.717	5.586	2.796	2.760
ORF27_48265	13.415	12.630	10.323	10.029	6.199	5.902	3.806	3.696	2.789	2.650	0.301	0.237
ORF29_49613	16.430	16.821	13.447	13.386	6.651	6.530	4.423	4.109	4.005	4.075	1.951	1.973
ORF30_51123	14.405	14.098	11.229	11.265	5.881	5.603	3.430	3.370	3.358	3.368	1.018	1.439
ORF32_52203	14.220	14.198	11.268	10.946	5.546	5.612	3.332	3.328	3.459	3.464	1.059	1.322
ORF33_53424	13.556	12.887	9.878	9.538	4.698	4.281	2.394	2.265	2.278	2.219	0.087	0.113
ORF34_55359	18.363	16.893	13.383	12.897	7.144	6.974	5.238	5.099	3.411	3.451	0.834	1.507
ORF37_58237	13.568	13.026	8.694	7.910	3.461	3.104	1.933	1.786	0.817	0.778	-1.184	-1.035
LANA78	7.576	7.426	8.244	8.484	6.695	6.558	6.712	6.573	6.085	6.115	3.953	3.578
ORF50_74850	2.135	9.397	8.235	7.991	0.631	0.417	-0.861	-0.903	-0.073	-0.080	-2.023	-1.985
ORF21_36500	15.672	15.734	12.868	12.935	6.890	6.685	5.339	5.264	4.162	4.363	2.737	3.132
ORF71_VFLIP_122381	7.222	7.197	7.846	8.000	6.147	5.861	5.978	5.823	4.060	4.093	2.214	1.982
LANA_124002	8.459	8.366	8.940	9.180	6.656	7.025	7.645	7.654	7.030	7.260	5.017	4.881
VIRF1K9_84086	10.975	10.970	10.996	10.657	8.717	8.501	7.270	7.328	4.957	5.050	2.216	2.405
VIRF2K11_92617	14.412	14.637	15.053	14.953	9.561	9.697	8.338	8.331	6.133	6.431	3.679	3.970
VIRF4K10_86975	13.556	13.694	12.961	12.675	7.809	7.717	6.293	6.403	3.942	4.086	2.037	2.145
ORF39_59355	11.768	11.230	9.686	9.923	5.049	4.956	2.896	3.049	3.387	3.417	0.779	0.833
ORF4_2360	9.174	9.124	9.244	9.126	8.214	8.274	7.292	7.508	4.645	4.484	2.799	3.085
ORF40_60631	12.293	11.906	13.118	13.158	7.597	7.464	5.778	5.732	5.476	5.519	3.192	3.248
ORF41_62161	11.372	11.548	11.530	11.205	7.251	6.761	5.219	5.128	5.191	5.080	2.611	2.453
ORF42_62756	18.196	18.210	15.142	15.748	9.482	9.051	7.340	7.333	5.755	5.573	3.442	3.263
ORF43_64036	17.755	19.580	15.302	15.123	9.576	9.382	7.574	7.524	5.845	5.725	3.061	3.294
ORF44_67024	11.463	11.895	11.823	11.371	8.801	8.817	7.253	7.386	5.610	5.547	3.119	3.099
ORF48_70402	17.679	15.665	13.786	12.943	7.201	7.030	5.678	5.792	4.798	4.832	2.867	3.000
ORF46_69318	13.414	13.600	10.281	9.975	3.477	3.451	2.315	2.341	1.064	1.159	-0.230	-0.069
ORF45_68227	10.008	10.038	8.933	8.783	3.315	3.256	2.212	2.095	1.112	1.211	-0.158	-0.174
ORF49_71851	11.288	11.426	11.123	11.027	6.805	6.470	5.126	5.113	5.009	5.204	2.991	3.059
ORF50_73509	10.063	10.029	9.853	9.658	2.634	2.699	1.776	1.794	2.171	2.333	0.514	0.721
K8_75170	12.516	12.321	11.433	11.045	6.533	6.122	4.747	4.552	5.381	5.339	3.452	3.544
ORF52_77128	15.889	16.474	12.057	11.248	6.149	6.116	4.114	3.952	1.952	1.836	0.458	0.523
ORF54_78056	16.924	16.292	12.978	13.289	7.352	6.898	5.878	5.901	4.741	4.779	3.073	3.176
ORF55_78956	17.290	16.655	15.200	14.579	8.406	7.814	7.120	6.937	5.764	5.754	4.111	3.651
ORF56_80963	15.485	15.843	14.770	14.870	7.919	7.907	6.537	6.546	5.684	5.761	3.995	3.933
ORF57_83216	11.476	11.456	7.902	7.375	0.262	-0.076	-0.504	-0.702	-0.950	-0.803	-2.041	-2.009
ORF58_95417	14.961	14.504	11.345	10.591	4.598	4.505	2.698	2.505	1.050	0.955	-1.344	-1.376
ORF59_96407	18.643	19.257	14.543	14.749	7.093	7.665	4.732	5.204	3.180	3.195	0.470	0.924
ORF6_5859	14.337	14.438	14.537	14.065	8.579	8.368	6.266	6.119	4.055	4.157	1.582	1.597
ORF60_97138	18.172	18.551	17.477	18.158	11.006	12.332	8.978	9.927	8.154	7.703	5.749	6.111
ORF62_100977	15.829	17.846	16.912	15.972	11.178	11.012	9.725	9.827	7.248	7.368	5.088	5.525
ORF29_54355	12.710	12.206	11.491	11.943	7.877	7.622	5.696	5.611	5.186	5.429	2.800	3.225
