# Supplementary material for: Mitochondrial protein, TBRG4, modulates KSHV and EBV reactivation from latency
Source: PLoS Pathog. 2022 Nov 23;18(11):e1010990. doi: 10.1371/journal.ppat.1010990 (PMC9683600; doi:10.1371/journal.ppat.1010990)
Supplement: S2 Table — (PDF) [file ppat.1010990.s008.pdf]

S2 Table. KSHV PCR array in KSHV-293 cells

	Delta Ct											
Target Name	NS-R1	NS-R2	NS-R3	siTBRG4-R1	siTBRG4-R2	siTBRG4-R3	NS-TPA/Na-R1	NS-TPA/Na-R2	NS-TPA/Na-R3	TBRG4-TPA/Na-R1	TBRG4-TPA/Na-R2	TBRG4-TPA/Na-R3
ORF9_13975	7.209	7.181	7.335	5.540	5.078	5.326	5.514	5.463	5.726	4.116	4.004	4.220
K1_622	8.570	8.508	8.608	7.101	7.079	7.144	5.954	5.908	6.037	6.182	5.995	5.956
K3_18883	5.041	5.038	5.206	3.923	3.804	3.943	4.173	4.083	4.215	3.530	3.319	3.279
K2_17821	4.637	4.611	4.510	2.381	2.364	2.269	2.338	2.313	2.453	1.269	1.109	1.109
ORF11_16083	5.986	5.909	5.980	4.109	4.030	4.142	4.242	3.975	4.251	3.071	2.764	2.947
ORF10_15288	6.598	6.558	6.460	4.655	4.559	4.530	4.880	4.903	4.967	3.668	3.480	4.059
ORF22_38336	6.159	6.016	5.868	4.223	4.005	3.938	4.377	4.267	4.305	2.939	2.738	2.672
ORF34_55680	6.620	6.653	6.452	4.420	4.232	4.328	3.217	3.235	3.422	1.814	1.746	1.892
ORF36_56709	5.577	5.554	5.522	3.645	3.547	3.558	2.847	2.745	2.885	1.407	1.313	1.385
ORF16_30403	7.581	7.605	7.745	6.186	6.151	6.475	6.607	6.475	6.583	5.184	4.973	5.036
LANA78	9.662	10.043	10.031	8.389	8.359	8.428	8.781	8.711	8.775	7.475	7.447	7.565
ORF50_74850	6.498	6.471	6.488	4.653	4.567	4.605	5.488	5.448	5.520	4.266	4.091	4.148
ORF21_36500	7.650	7.656	7.516	5.437	5.398	5.295	5.585	5.317	5.459	4.081	3.796	3.754
ORF71_VFLIP_122381	7.890	7.805	7.708	6.662	6.526	6.470	7.060	7.103	7.289	6.267	6.217	6.119
ORF73_LANA_124002	10.035	9.878	9.933	8.945	8.621	8.541	8.767	8.713	8.912	7.876	7.789	7.896
LANA78	9.861	9.849	9.854	8.350	8.272	8.294	8.794	8.753	8.721	7.555	7.463	7.462
ORF17_32510	6.720	6.720	6.728	4.691	4.779	4.790	4.888	4.883	5.011	4.082	3.788	3.359
VGPCR_129945	9.105	9.227	9.017	7.340	7.321	7.489	8.430	8.490	8.658	7.344	7.159	7.249
VGPCR_130126	8.772	8.982	8.897	7.154	7.159	6.962	8.309	8.189	8.537	7.129	7.001	6.920
ORF75_130749	6.801	6.763	6.830	5.869	5.760	5.868	6.404	6.396	6.521	6.304	6.142	6.173
K15_131117	7.305	7.326	7.308	6.188	6.202	6.185	6.462	6.427	6.565	6.294	6.192	6.203
ORF65_112376	7.961	7.707	7.752	6.366	6.241	6.654	7.286	7.224	7.803	7.268	6.438	6.978
K3_18883	5.187	5.217	5.073	3.785	3.716	3.769	4.055	4.076	4.127	3.444	3.295	3.241
K4_21778	3.565	3.493	3.462	2.029	1.745	1.716	2.403	2.361	2.385	1.195	1.078	1.121
K7_28624	7.333	7.088	7.164	5.971	5.896	6.167	6.413	6.455	6.644	5.136	5.061	5.084
K5_26145	6.291	6.228	6.222	4.161	4.157	4.179	3.212	3.138	3.275	2.018	1.815	1.880
ORF17_31509	3.988	4.133	4.011	2.404	2.354	2.515	3.083	2.991	3.063	1.988	1.884	1.688
ORF18_32935	6.511	6.564	7.460	4.524	4.393	4.478	5.009	5.025	5.099	3.675	3.321	3.340
ORF63_103119	8.891	8.686	8.725	6.776	6.804	6.839	6.617	6.588	6.865	5.199	4.916	5.025
ORF64_111231	7.240	7.354	7.280	5.823	5.812	5.409	5.379	5.300	5.435	4.032	3.867	3.821
ORF66_114453	8.829	8.903	8.785	7.068	6.961	7.039	7.576	7.463	7.840	6.522	6.233	6.522
ORF67_113980	9.356	9.230	9.498	7.487	7.311	7.644	8.170	8.034	8.126	6.998	6.873	6.790
ORF68_115939	7.836	7.883	8.014	6.344	6.253	6.292	6.919	6.910	7.211	5.922	5.734	5.764
ORF69_117120	6.721	6.808	6.876	5.384	5.276	5.394	6.103	5.994	6.301	5.385	5.129	5.181
ORF7_8384	8.213	8.209	8.030	6.178	6.090	5.926	6.420	6.253	6.518	5.109	4.863	4.891
ORF70_20979	7.267	7.327	7.351	6.029	5.844	5.952	5.483	5.410	5.659	4.890	4.830	4.749
VCYC_123054	8.470	8.430	8.461	7.497	7.348	7.341	7.682	7.552	7.641	6.571	6.521	6.553
ORF8_10765	7.488	7.317	7.575	5.554	5.545	5.587	6.092	6.174	6.409	4.872	4.748	4.963
ORF19_34089	7.100	7.246	6.773	4.730	4.766	4.582	5.133	5.195	5.320	3.621	3.459	3.477
ORF20_34848	7.435	7.439	7.396	5.962	5.855	5.486	6.117	6.127	6.288	4.738	4.596	4.717
ORF23_39786	6.706	6.690	6.017	4.199	4.259	4.289	4.461	4.545	4.765	3.224	2.992	2.960

ORF24_41630	7.276	7.089	7.113	5.297	5.130	5.057	5.352	5.324	5.278	3.700	3.649	3.555
ORF25_45767	7.230	7.319	7.251	5.373	5.093	5.089	5.850	5.630	5.678	4.018	3.846	3.979
ORF27_48265	3.627	3.605	3.629	2.080	1.981	2.043	2.329	2.416	2.350	1.118	1.019	0.942
ORF29_49613	8.535	8.559	8.354	7.007	6.862	6.916	7.059	7.053	7.198	5.731	5.593	5.612
ORF30_51123	8.479	8.522	8.352	6.328	6.226	6.160	6.149	6.046	6.198	4.428	4.323	4.637
ORF32_52203	8.038	7.865	7.642	5.627	5.518	5.560	5.495	5.503	5.726	4.130	4.027	4.088
ORF33_53424	5.335	5.410	5.438	3.673	3.705	3.757	3.761	3.931	3.968	2.938	2.722	2.654
ORF34_55359	8.171	8.088	8.177	5.985	5.932	5.890	5.715	5.678	5.854	4.457	4.123	4.256
ORF37_58237	4.027	4.000	4.012	2.519	2.344	2.407	1.986	1.980	1.940	0.942	0.796	0.685
LANA78	9.543	9.626	9.718	8.428	8.228	8.390	8.683	8.575	8.581	7.524	7.363	7.338
ORF50_74850	6.445	6.428	6.516	5.535	5.390	4.634	5.506	5.488	5.504	4.323	4.072	4.323
ORF21_36500	7.718	7.682	7.628	5.494	5.375	5.354	5.562	5.412	5.627	3.910	3.640	4.042
ORF71_VFLIP_122381	7.703	7.685	7.687	6.562	6.486	6.514	7.177	7.107	7.196	6.210	6.008	6.088
LANA_124002	10.026	9.971	9.909	8.647	8.595	8.722	7.998	7.999	8.892	8.000	7.642	7.815
VIRF1K9_84086	7.180	7.203	7.114	5.487	5.476	5.361	4.882	4.916	5.137	3.998	3.765	3.979
VIRF2K11_92617	8.151	8.191	8.222	6.507	6.257	6.383	6.376	6.387	6.617	5.216	4.906	5.161
VIRF4K10_86975	6.012	5.871	5.834	4.119	4.061	3.975	5.163	5.169	5.105	3.926	3.725	3.701
ORF39_59355	5.920	5.763	5.722	4.283	4.306	4.219	4.672	4.622	4.616	4.010	3.771	3.590
ORF4_2360	8.110	8.171	7.970	7.198	7.202	7.088	6.476	6.600	6.648	6.933	6.808	6.776
ORF40_60631	8.905	9.009	8.953	6.992	6.892	6.903	7.077	7.011	7.119	5.549	5.367	5.583
ORF41_62161	7.145	7.198	6.961	5.324	5.231	5.208	5.787	5.666	5.815	4.556	4.272	4.407
ORF42_62756	7.017	7.107	7.037	6.130	5.717	5.759	6.810	6.722	6.769	5.938	5.704	5.764
ORF43_64036	8.365	8.397	8.329	6.899	6.775	6.782	7.615	7.543	7.632	6.669	6.506	6.552
ORF44_67024	7.570	7.536	7.554	5.808	5.761	5.733	6.504	6.487	6.562	5.670	5.407	5.392
ORF48_70402	8.212	8.254	8.294	6.468	6.359	6.391	6.868	6.806	6.956	5.564	5.464	5.559
ORF46_69318	6.677	6.826	6.477	4.752	4.652	4.727	3.863	3.796	4.063	2.668	2.562	2.772
ORF45_68227	5.225	5.186	5.107	3.860	3.733	3.714	3.242	3.173	3.392	2.374	2.145	2.238
ORF49_71851	8.325	8.255	8.377	7.007	6.989	6.909	7.717	7.682	7.876	6.837	6.640	6.724
ORF50_73509	7.915	7.784	7.677	6.164	5.945	6.072	6.729	6.555	6.583	5.530	5.387	5.406
K8_75170	9.669	9.714	9.280	7.826	7.702	7.763	8.091	7.954	8.073	7.058	6.970	6.947
ORF52_77128	5.008	4.847	4.930	3.455	3.378	3.465	5.240	4.787	4.421	3.116	2.890	3.048
ORF54_78056	5.877	6.028	5.878	3.490	3.446	3.374	3.763	3.626	3.707	2.239	2.042	2.192
ORF55_78956	7.418	7.331	7.197	5.768	5.898	5.814	6.202	6.085	6.181	5.141	4.929	4.821
ORF56_80963	9.059	9.084	9.136	7.085	6.886	6.860	6.956	6.966	7.031	5.539	5.365	5.480
ORF57_83216	3.815	3.818	3.919	1.911	1.794	1.830	1.142	1.063	1.213	0.022	-0.223	-0.295
ORF58_95417	3.860	3.756	3.732	2.099	1.984	1.967	1.842	1.751	1.932	0.602	0.390	0.382
ORF59_96407	7.552	7.530	6.947	5.613	5.390	5.180	4.786	4.716	5.169	3.505	3.253	3.549
ORF6_5859	5.969	5.986	5.896	3.970	3.870	3.897	4.602	4.508	4.633	3.289	3.124	3.182
ORF60_97138	11.726	11.532	10.542	8.799	8.439	7.975	7.743	7.436	7.647	5.767	5.555	5.030
ORF62_100977	8.318	8.225	8.168	6.294	6.257	6.213	6.608	6.447	6.671	4.933	4.781	4.739
ORF29_54355	9.000	8.866	9.162	7.129	6.849	6.982	6.961	6.935	6.960	5.478	5.317	5.444
