# Supplementary material for: Mitochondrial protein, TBRG4, modulates KSHV and EBV reactivation from latency
Source: PLoS Pathog. 2022 Nov 23;18(11):e1010990. doi: 10.1371/journal.ppat.1010990 (PMC9683600; doi:10.1371/journal.ppat.1010990)
Supplement: S3 Table — (PDF) [file ppat.1010990.s009.pdf]

S3 Table. Primers for real-time PCR

Name	Forward (5'-3')	Reverse (5'-3')
TBRG4	TCCCCAACTTCAACAGCC	AGTTCCAGCCACTCATAGAATG
K8.1	AAAGCGTCCAGGCCACCACAGA	GGCAGAAAATGGCACACGGTTAC
ORF39	GGTTTCCCCTGCTACTTCAA	CATGCTTGGCCCGATATAC
ORF57	TGGACATTATGAAGGGCATCCTA	CGGGTTCGGACAATTGCT
ORF52	AAATCGAAGCCAGGGTCAGG	CTCCTCTTCGTCGCCTGTTATTG
Kaposin	GTTGCAACTCGTGTCCTGAA	GGCTTAACGGTGTTTGTGG
LMP1	CAGTCAGGCAAGCCTATGA	CTGGTTCCGGTGGAGATGA
BMRF1	TTAGAAACCTTGCCTACGGG	AAAATTGCAGGGAAGCCTGC
BALF2	CCGTGGGTCATGTAGAAACTG	ACACCGATATAATGCCAGCC
BLLF1	TGGGATGTAGACAAGTTACGCCT	TGCTGACCCTTCTGCTGCT
Kaposin	GGTGTTTGTGGCAGTTCATG	AACTCGTGTCCTGAATGCTAC
MT-ND1	GGCTATATACAACTACGCAAAGGC	GGTAGATGTGGCGGGTTTTAGG
MT-ND2	CACAGAAGCTGCCATCAAGTA	CCGGAGAGTATATTGTTGAAGAG
MT-ND3	AGAAAAATCCACCCCTTACGAGT	TGGAGAAAGGGACGCGG
MT-ND4	CCCTCGTAGTAACAGCCATTCTC	CGACTGTGAGTGCGTTCGTAGT
MT-ND5	ACATCTGTACCCACGCCTTC	TATGTTTGCGGTTTCGATGA
MT-ND6	CACAGCACCAATCCTACCTCCA	GCGATGGCTATTGAGGAGTATCC
MT-COX1	TCTCAGGCTACACCCTAGACCA	ATCGGGGTAGTCCGAGTAACGT
MT-COX2	TGCCCGCCATCATCCTA	TCGTCTGTTATGTAAAGGATGCGT
MT-COX3	CCAATGATGGCGCGATG	CTTTTTGGACAGGTGGTGTGTG
MT-ATP6	CCAATAGCCCTGGCCGTAC	GCTTCCAATTAGGTGCATGA
MT-ATP8	CAACTAAAAATATTAAACACAA	CGTTCATTTTGGTTCTCAGG
B-Actin	AAGACCTGTACGCCAACACA	AGTACTTGCGCTCAGGAGGA
